# Chimeric Nanocurved Materials Reprogram Receptor Signaling to Induce Apoptosis via a Curvature-Sensing Protein

**DOI:** 10.1021/acsnano.6c00463

**Published:** 2026-08-05

**Authors:** Shota Yamamoto, Naoki Fukata, Wipakorn Jevasuwan, Moritoshi Sato, Jun Nakanishi

**Affiliations:** † Research Center for Macromolecules & Biomaterials, National Institute for Materials Science (NIMS), 1-1 Namiki, Tsukuba, Ibaraki 305-0044, Japan; ‡ Research Center for Materials Nanoarchitectonics, National Institute for Materials Science (NIMS), 1-1 Namiki, Tsukuba, Ibaraki 305-0044, Japan; § Graduate School of Arts and Sciences, 68394 The University of Tokyo, 3-8-1 Komaba, Meguro-ku, Tokyo 153-8902, Japan; ∥ Kanagawa Institute of Industrial Science and Technology, 3-2-1 Sakado, Takatsu-ku, Kawaski-shi, Kanagawa 213-0012, Japan; ⊥ Graduate School of Advanced Science and Engineering, Waseda University, 3-4-1 Okubo, Shinjuku-ku, Tokyo 169-8555, Japan; # Graduate School of Advanced Engineering, Tokyo University of Science, 6-3-1 Niijuku, Katsushika-ku, Tokyo 125-8585, Japan; ¶ Research Center for Autonomous Systems Materialogy (ASMat), Institute of Integrated Research (IIR), Institute of Science Tokyo (Science Tokyo), 4259 Nagatsuta-cho, Midori-ku, Yokohama, Kanagawa 226-8501, Japan

**Keywords:** nanostructures, apoptosis, epidermal growth factor, BAR protein, cancer cell, mechanobiology

## Abstract

The nanoscale curvature of cell membranes plays a regulatory role in cell activity and response. This paper reports chimeric nanocurved materials functionalized with EGF that are capable of reprogramming growth factor signaling via a curvature-sensing protein. We achieved this by integrating epidermal growth factor (EGF) onto the surface of various nanostructured materials, which were exposed to the apical surface of cell membranes. These materials selectively triggered apoptosis, in contrast to flat EGF-modified surfaces. Serial variation of the nanoscopic parameters of the EGF-functionalized nanomaterials confirmed that the synergistic combination of the nanocurved material-mediated stimulation and receptor activation is critical for apoptosis induction. RNA-seq revealed a shift toward a pro-apoptotic gene expression profile, characterized by the suppression of survival-related pathways and enhancement of apoptosis-associated signals. Furthermore, gene editing experiments demonstrated that the nanocurved materials recruit the curvature-sensing protein PACSIN2, which in turn modulates the signaling cascade to induce apoptosis. Our findings show that chimeric nanocurved EGF-functionalized materials reprogram EGFR signaling from pro-survival to pro-apoptotic outcomes via an endocytosis-independent pathway mediated by PACSIN2. This mechanism underscores the potential of engineered biointerfaces to control receptor-mediated cell fate and suggests opportunities for patch-based anticancer therapies.

## Graphical abstract

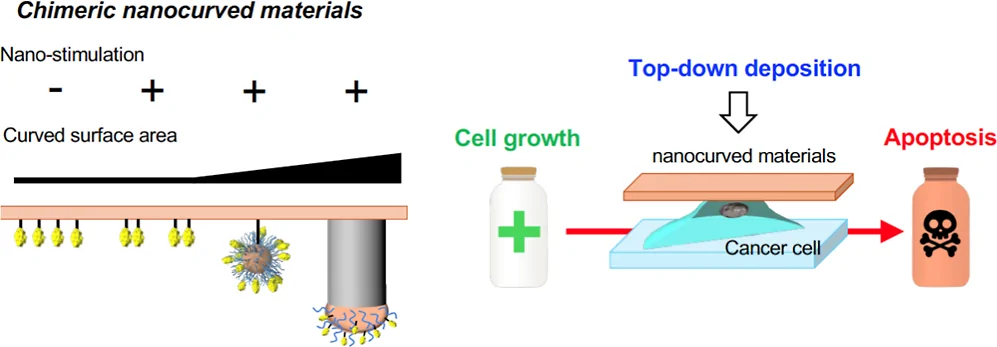

## 1 Introduction

The plasma membrane is a dynamic and flexible structure that serves as a platform for transport, receptor–ligand interactions, intercellular communication, and biochemical reactions.^1,2^ Cells continuously respond to mechanical and biochemical stimulation from the extracellular matrix, such as collagen and proteoglycans, leading to membrane remodeling and functional adaptation.^3^ Understanding how the membrane structure influences cellular function is essential for medical applications, including elucidating disease mechanisms and developing cancer treatments.^4^ The combination of material fabrication techniques and imaging technologies has enabled the investigation of cell membrane structure–function relationships at the nanoscale level.^5–7^ Nanotopographic materials or nanofibers have been used to study cellular alignment and polarity formation through contact guidance mechanisms.^8^ Also, molecular-scale nanopatterned surfaces elucidated the necessary distance for the receptor clustering and focal adhesion maturation.^9,10^ Recently, subcellular-size vertical nanostructures, such as silicon nanowires (SiNWs) and nanoneedles, were shown to manipulate plasma membrane curvatures. Interestingly, such artificial membrane curvature generation can trigger cytoskeletal rearrangement and enhanced endocytosis through the recruitment of bin/amphiphysin/rvs (BAR) proteins, established membrane curvature sensors.^11^ By mimicking the in vivo scaffold environment, nanowires facilitate cancer cell migration within a 3D matrix through curved adhesion.^12^ These results strongly suggested that physical stimulation of the plasma membranes can solely rearrange the membrane compositions and associated proteins to induce signal reprogramming even in the absence of biochemical cues such as proteins or ligands. Thus, integrating nanostructures with biochemical factors, two stimuli previously considered independently, could give rise to previously inaccessible cellular functions.

A representative example of this concept is the conjugation of the epidermal growth factor (EGF) to nanomaterials. We demonstrated this phenomenon by showing that immobilizing EGF onto nanoparticles confined its activity to nanoscale membrane rafts (80–100 nm), thereby converting EGFR signaling from proliferative to an apoptotic response.^13–15^ This mechanism highlights the importance of particle-mediated signal condensation within membrane rafts in driving the unique apoptotic activity of EGF-nanoparticle conjugates. In contrast, EGF immobilized on flat cell scaffolds promotes cell growth and differentiation by sustaining EGFR activity while preventing endocytosis.^16,17^ Furthermore, nanoparticles exceeding 500 nm in diameter bearing immobilized EGF were not internalized by the cells and induced cell proliferation.^18^ These findings raise the hypothesis that EGF immobilization on materials modulates cellular signaling through a combination of biochemical and nanocurved material-mediated stimulation, leading to phenotypic outcomes such as cell proliferation or death.

To investigate this hypothesis and explore how nanoscale ligand presentation and nanocurved material-mediated stimulation cooperatively modulate cellular responses, we designed chimeric nanocurved materials (Figure 1). Here, the nanocurved materials are termed “chimeric” because they combine nanostructures with the biochemical ligand EGF. By applying them directly to cancer cell membranes, we aimed to determine how cells integrate these stimuli and whether nanoscale control of receptor activation could alter cell fate. To gain deeper mechanistic insight, we further employed transcriptomic profiling by RNA-sequencing (RNA-seq) and functional validation using CRISPR-Cas9–mediated gene editing. This strategy provides a framework to uncover how the interplay between ligand presentation and nanoscale material features reprograms EGFR signaling, while also highlighting the potential of such engineered biointerfaces as platforms for next-generation therapeutic development.

**Figure 1:**
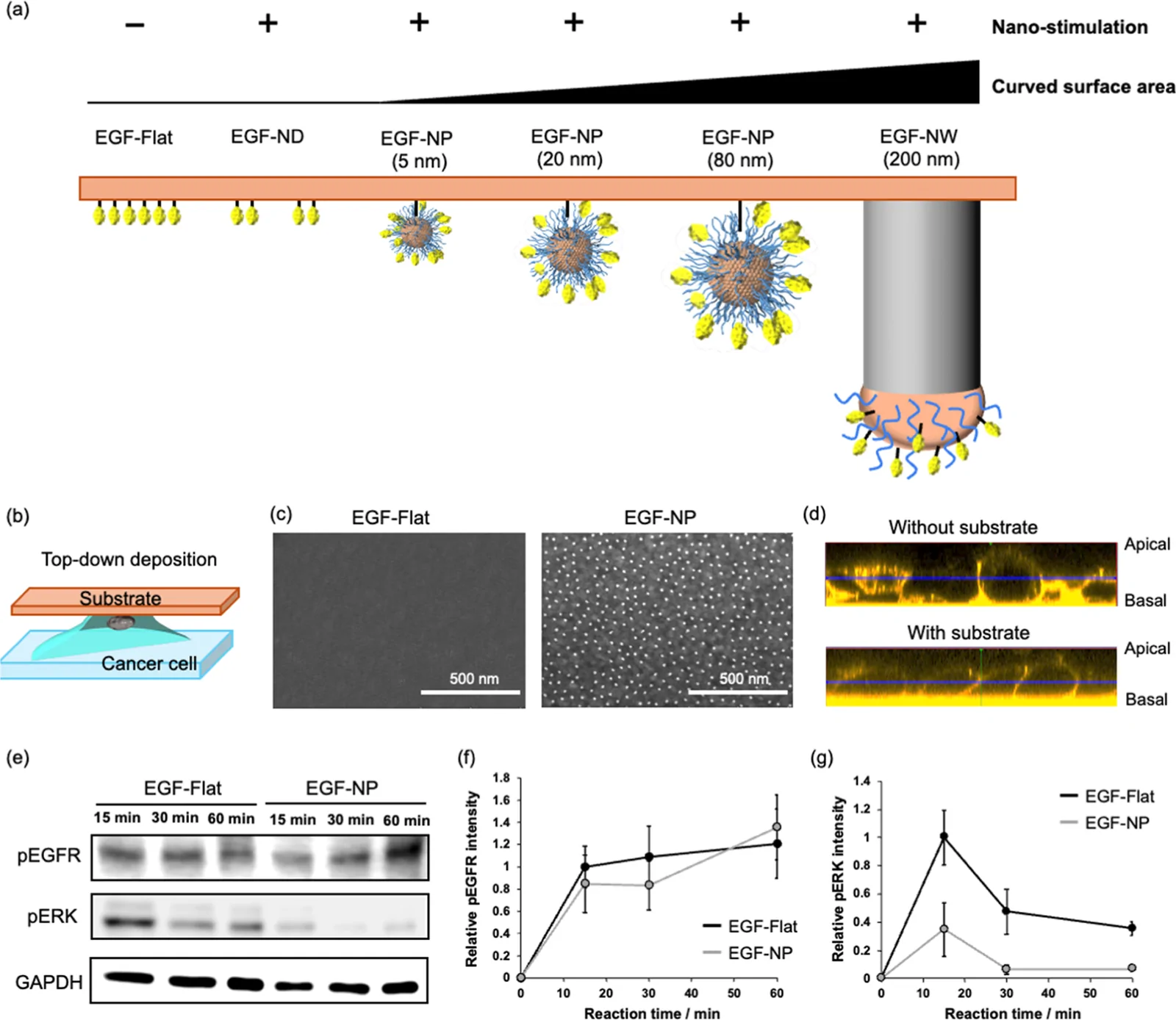
Surface engineering and characterization of chimeric nanocurved EGF-installed materials. (a) Strategic surface design of EGF-Flat, EGF-ND, EGF-NP and EGF-NW and the cellular stimulation induced by each material. (b) Experimental setup for applying each surface from the apical side of cancer cells. (c) SEM image of EGF-Flat and EGF-NP. (d) Confocal images of A431 cells with and without contact with EGF-NP. Fluorescence imaging shows the cellular morphology after 4 h incubation with EGF-NP (top panel) compared to cells without surface contact (bottom panel). (e) Representative Western blotting results. Blots show protein expression in A431 cells after 15, 30, and 60 min of exposure to EGF-Flat or EGF-NP. Phosphorylated EGFR (pY1068) and phosphorylated ERK1/2 (Thr202/Tyr204) were detected using specific antibodies, with GAPDH used as a loading control. (f,g) Time-course analysis of phosphorylation levels of EGFR (f) and ERK (g) in A431 cells treated with EGF-Flat (black) or EGF-NP (gray). The data are shown as the mean ± SD from at least three independent experiments. Statistical differences were evaluated by Student’s *t*-test.

## 2 Results

### 2.1 Pharmacology- and Materials-Based Endocytosis Inhibition of EGF-Nanoconjugates

To establish a benchmark of our EGF-nanomaterial-based studies, we first addressed whether endocytosis of EGF conjugated to gold nanoparticle (EGF-AuNP; Figure S1, Supporting Information) was essential for apoptosis induction in HeLa cells. For this, we used dynasore, a membrane-permeable dynamin inhibitor, known to block endocytosis via preventing the fission of coated vesicles.^19^ The distribution of gold nanoparticles in cells with and without dynasore treatment is visualized in dark-field microscopic images (Figure S2a, Supporting Information). In untreated cells, most gold nanoparticles disappeared from the cell surface within 20 min of addition, consistent with their uptake via endocytosis. In contrast, in dynasore-treated cells, a large number of gold nanoparticles remained on the plasma membrane even after 20 min, indicating that cellular internalization was effectively inhibited by the drug. Subsequently, apoptosis induction assays were performed. HeLa cells were treated with EGF-conjugated gold nanoparticles, and apoptotic cells were evaluated by Annexin V staining. Dynasore treatment reduced the proportion of Annexin V–positive cells by approximately 30% compared to untreated cells. However, cells still underwent apoptosis even when endocytosis of both receptors and AuNPs was inhibited (Figure S2b, Supporting Information). Previous studies have suggested that apoptosis induction by EGF-conjugated nanoparticles results from unique membrane trafficking following endocytosis.^20,21^ However, our results demonstrate that endocytosis is not required for EGF-nanoparticle-mediated apoptosis induction.

Encouraged by these findings, we moved to more stringent experimental condition in which EGF molecules were immobilized on the solid substrate surface (Figure 1a). The functionalized surface was placed at the apical side of cancer cells, thereby enabling physical stimulation of EGFR while excluding endocytosis by the bioconjugation (Figure 1b). Moreover, this strategy allowed us to manipulate two distinct nanofeature stimuli either simultaneously or individually by changing the nanofeatures on the solid surface, such as conjugating AuNPs with different diameters through biotin–avidin chemistry and lithographically prepared nanowires. Here, we used two types of EGF-installed nanostructured materials. EGF-NP is a flat substrate with immobilized EGF-conjugated gold nanoparticles designed to simultaneously provide biochemical stimulation and nanocurved material-mediated stimulation (Figures S3a,b and S4, Supporting Information). EGF-Flat, in which EGF is immobilized homogeneously on a flat gold substrate, induces widespread receptor activation due to the uniform distribution of EGF (Figure S3c, Supporting Information). At first, we observed the distribution of gold nanoparticles on the surface of EGF-NP using scanning electron microscopy (SEM). Successful conjugation of AuNPs was confirmed by SEM, which revealed uniformly distributed nanoparticles on the surface of the EGF-NP substrate, whereas no immobilized particles were observed on the EGF-Flat surface, where only proteins were bound (Figure 1c). To visualize the interaction of cells with the surfaces, A431 cells stained with a fluorescent membrane dye after apical contact with the substrates were examined. Cells without substrate application exhibited variable heights, whereas those exposed to the substrate maintained a uniform height, indicating consistent apical contact across the cell population (Figure 1d). To investigate the cellular signaling response to each surface, we analyzed EGFR (Y1068) and ERK phosphorylation using Western blotting. The EGFR phosphorylation pattern induced by EGF-NP was comparable to that induced by EGF-Flat (Figure 1e,f). Notably the phosphorylation of ERK, a key downstream effector of the EGFR signaling cascade, was reduced to approximately half the level observed with EGF-Flat (Figure 1e,g). Cells cultured on flat substrates with immobilized EGF have been shown to promote proliferation through sustained activation of ERK, as activated EGFR remains on the plasma membrane without undergoing endocytosis.^22^ In contrast, our results demonstrate a distinct outcome when EGF-Flat is applied from the apical side of the cells. Although EGF-NP induces EGFR activation to a similar extent as EGF-Flat, it results in downregulation of ERK activation. Next, we quantified the surface density of EGF immobilized on EGF-NP and EGF-Flat using a direct ELISA with an anti-EGF antibody.^23^ The estimated surface densities of immobilized EGF were 4.4 × 10^4^ and 1.3 × 10^4^ molecules μm^–2^ for EGF-Flat and EGF-NP, respectively. Despite this difference in ligand density, the levels of phosphorylated EGFR were comparable between the two substrates (Figure 1f). These results suggest that nanocurved material-mediated stimulation introduced by nanoparticle-based nanostructures modulates downstream EGFR signaling. Furthermore, our strategy prevented endocytosis by immobilizing EGF onto particles or substrates. Endocytosis contributes to receptor desensitization and active signal transduction; therefore, its inhibition may lead to downregulation of downstream signaling pathways.^24^ Our findings reveal that precise nanoscale regulation of spatial cues and nanocurved material-mediated stimulation of EGFR at the plasma membrane collectively modulate intracellular signaling pathways.

### 2.2 Nanoscopic EGF Exposure through AuNPs Induces Cellular Apoptosis

We further investigated whether nanocurved material-mediated stimulation and receptor activation alter cellular fate by modulating downstream signaling. PEG-Flat, EGF-Flat, PEG-NP, or EGF-NP was applied to A431 cells, and apoptosis was assessed after 4 h using caspase-3/7 activity as an indicator.^25^ In this experimental setup, the substrate was applied to the apical side of the cells. It has been reported that excessive mechanical compression of cells induces cell death, primarily through apoptosis or necrosis, depending on the magnitude and duration of the applied stress.^26^ Therefore, we evaluated the effect of mechanical stress generated by our system on apoptosis. A431 cells treated with PEG-Flat, in which PEG2k was immobilized on a gold substrate, did not exhibit caspase-3/7 activation, even after 4 h of exposure. This result strongly suggests that mechanical stress has a negligible effect and can be effectively excluded as a contributor to apoptosis in A431 cells. We then applied EGF-Flat to the apical side of the cells for 4 h and evaluated caspase-3/7 activation. Similar to PEG-Flat, EGF-Flat scarcely activated caspase-3/7 (Figures 2a,b and S5, Supporting Information). Next, we conducted similar experiments using EGF-NP. The nanostructured substrates with immobilized nanoparticles significantly induced caspase-3/7 activation. In contrast, PEG-NP lacking EGF did not induce apoptosis. In our system, EGF-NP induced apoptosis despite EGFR activation being confined to the plasma membrane. Our results indicate that combining nanocurved material-mediated stimulation, which deforms the plasma membrane with EGF signaling can shift cellular response from survival to apoptosis. To further investigate the role of nanocurved material-mediated stimulation, we prepared EGF-NP using gold nanoparticles of two different diameters: 5 and 80 nm. Surfaces prepared with 5 nm particles did not induce caspase-3/7 activation, whereas those with 80 nm particles significantly promoted apoptosis (Figures 2c,d and S6, Supporting Information). These findings suggest that 5 nm particles provided EGF stimulation but failed to induce sufficient membrane deformation, while 80 nm particles delivered both biochemical and nanocurved material-mediated stimuli, thereby switching EGF signaling toward apoptosis. Notably, a threshold level of nanocurved material-mediated stimulation, achievable with particles larger than 20 nm, is required to trigger an apoptotic response. These results suggest that cancer cells activate EGF receptors through the nanostructures and undergo apoptosis by remodeling their plasma membrane architecture in response to these nanostructures. To further assess the synergy between biochemical and nanocurved material-mediated stimulation, both stimulations were applied. The EGF-NP induced membrane deformation specifically at EGF-functionalized sites, restricting EGFR activation to these localized membrane regions. Therefore, caspase-3/7 activation was examined following the independent application of nanocurved material-mediated stimulation via PEGylated nanoparticles and biochemical stimulation via soluble EGF (Figure 2e). A431 cells were exposed to these surfaces in the presence of 1 ng/mL soluble EGF. No apoptosis was observed under these conditions (Figure 2f,g), a result comparable to that observed with soluble EGF treatment alone, which provided only biochemical stimulation. In contrast, EGF-NP effectively activated caspase-3/7. These results suggest that both the nanocurvature features and biochemical ligand exposure are critical for the apoptosis induction. Previous reports have shown that the nanodomain presentation of bioactive ligands such as cRGD and fibronectin can elicit distinct cellular responses compared with uniformly immobilized ligands.^27,28^ It was hypothesized that EGF immobilized on nanoparticles might also drive cancer cells toward apoptosis by spatially creating localized stimulation at the membrane. To modulate the particle density, biotinylated substrates were prepared by mixing 50 μM of biotinylated PEG2k disulfide and monomethyl PEG2k disulfide in molar ratios of 1:0, 1:1, and 1:9. Biotinylated EGF nanoparticles were then immobilized on these substrates to obtain EGF-NP with different modification densities. When these nanostructures were applied to cancer cells, EGF-NP at 100% and 50% modification density induced caspase-3/7 activity, while EGF-NP at 10% density did not (Figure S7, Supporting Information). These results suggest that apoptosis induction by EGF nanostructures requires membrane deformation and spatially localized biochemical stimulation.

**Figure 2:**
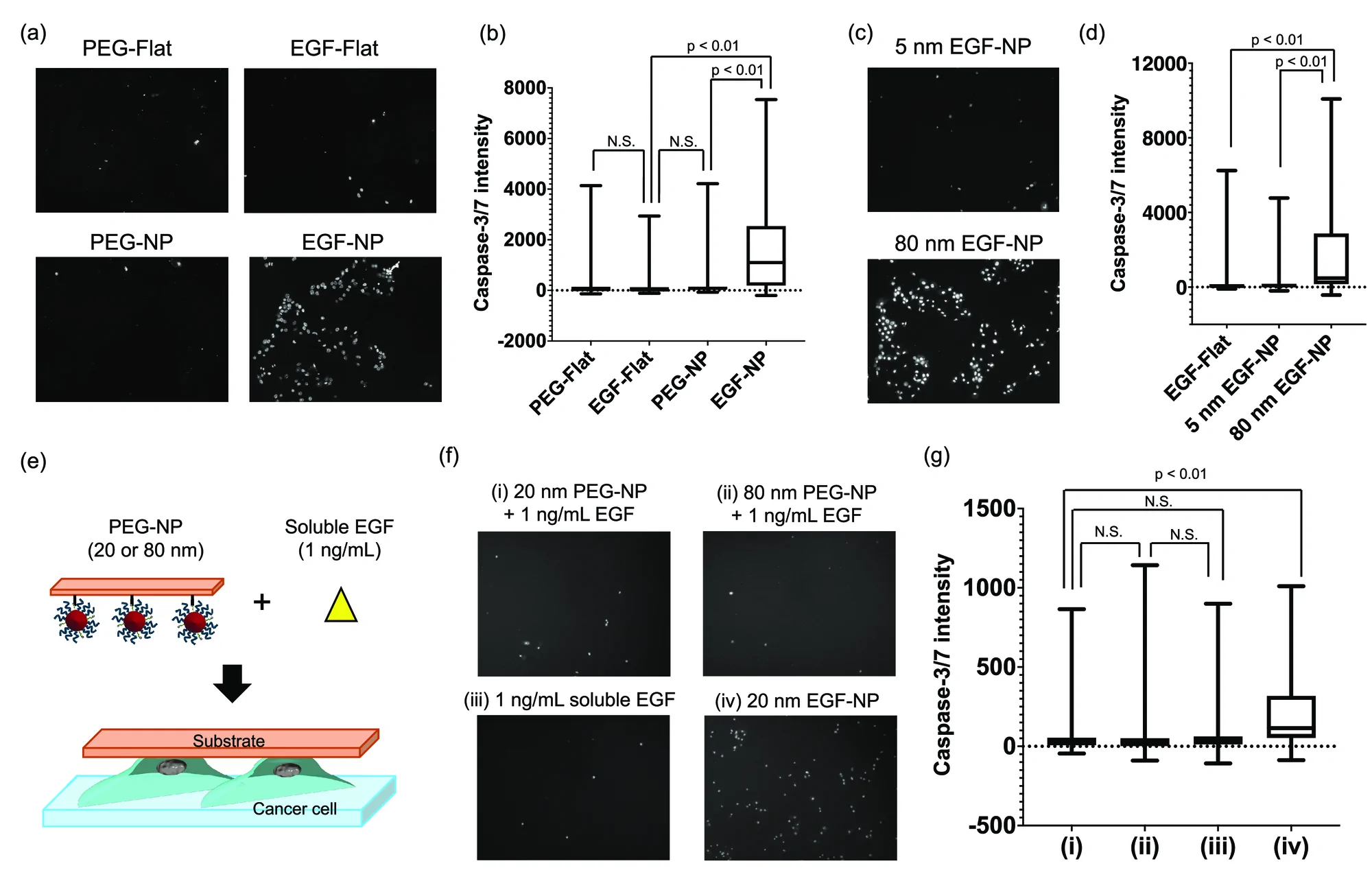
Apoptosis induction by chimeric nanocurved materials. Apoptosis was evaluated using caspase-3/7 activity using the CellEvent caspase-3/7 green detection reagent. A431 or MDA-MB468 cells were incubated with the indicated surfaces for 4 h and analyzed using fluorescence microscopy. (a,b) Caspase-3/7 activity was measured in A431 cells incubated for 4 h with PEG-Flat, EGF-Flat, PEG-NP, or EGF-NP. (c,d) Caspase-3/7 activity was evaluated in A431 cells treated with EGF-NP prepared using 5 or 80 nm gold nanoparticles, to examine the effect of particle size. (e–g) Combinatorial effect of nanocurved material-mediated and biochemical stimulation on apoptosis induction. Caspase-3/7 activity in A431 cells after 4 h incubation with 20 or 80 nm PEG-NP in the presence of 1 ng/mL soluble EGF. The data are shown as the mean ± SD from at least three independent experiments. Statistical differences were evaluated by Student’s *t*-test.

### 2.3 RNA-Seq Analysis Demonstrated that EGF-NP Reprograms Cellular Signaling toward Apoptosis

To investigate the unique apoptosis-inducing mechanism triggered by the EGF-NP, a comprehensive transcriptomic analysis was performed using RNA-seq with a next-generation sequencer (Figure 3). Gene expression changes were analyzed in EGF-NP-treated cells, using EGF-Flat which exhibits no pro-apoptotic activity as a reference. Although EGF is conventionally known to promote cell survival and proliferation through EGFR signaling, RNA-seq analysis revealed an unexpected transcriptional response upon treatment with the EGF-NP. Specifically, multiple pro-survival pathways,^29–31^ including NF-κB, MAPK, and JAK-STAT signaling, were significantly downregulated (Figure 3a,b). Simultaneously, genes associated with the intrinsic apoptotic pathway, such as Bad^32^ (Figure 3c), were markedly upregulated. Although the PI3K/AKT signaling pathway was not fully downregulated, a comprehensive analysis revealed a selective reduction in AKT1 expression, whereas AKT2 remained unchanged. In addition, GAB1, a critical adaptor linking EGFR to PI3K/AKT activation, was downregulated. These changes suggest that AKT signaling is functionally impaired at multiple levels. This impairment likely contributed to the upregulation of pro-apoptotic genes such as Bad.^33^ These findings suggest that EGF immobilized on the material surface induces noncanonical EGFR signaling, possibly mediated by nanocurved material-mediated stimulation. Such atypical stimulation overrides normal proliferative cues and triggers a cellular response that shifts cell fate toward apoptosis. Furthermore, the downregulation of upstream immune and cytokine-related signaling pathways including TNF signaling, IL-17 signaling, and cytokine–cytokine receptor interaction contributed to the attenuation of NF-κB and JAK-STAT activity, exacerbating the loss of pro-survival signaling.^34–36^ Notably, these pathways are not independent but interconnected through extensive crosstalk (Figure 3b). NF-κB and STAT3 coregulate the transcription of the antiapoptotic gene of BCL2L1 (encoding Bcl-xL).^37^ The concurrent suppression of both pathways synergistically impaired the capacity of the cells to resist apoptosis. The concurrent downregulation of Bcl-xL and upregulation of Bad supports this hypothesis. The findings indicate that EGF-functionalized materials do not simply activate classical EGFR signaling but orchestrate the complex reprogramming of cellular signaling networks, ultimately leading to programmed cell death. This likely reflects the well-established principle that ligand presentation significantly affects cell behavior, with material-bound growth factors eliciting distinct responses compared to their soluble counterparts. These results raise the question of whether this apoptosis-inducing signaling is driven by nanocurved material-mediated stimulation, localized EGF stimulation, or a combination of both.

**Figure 3:**
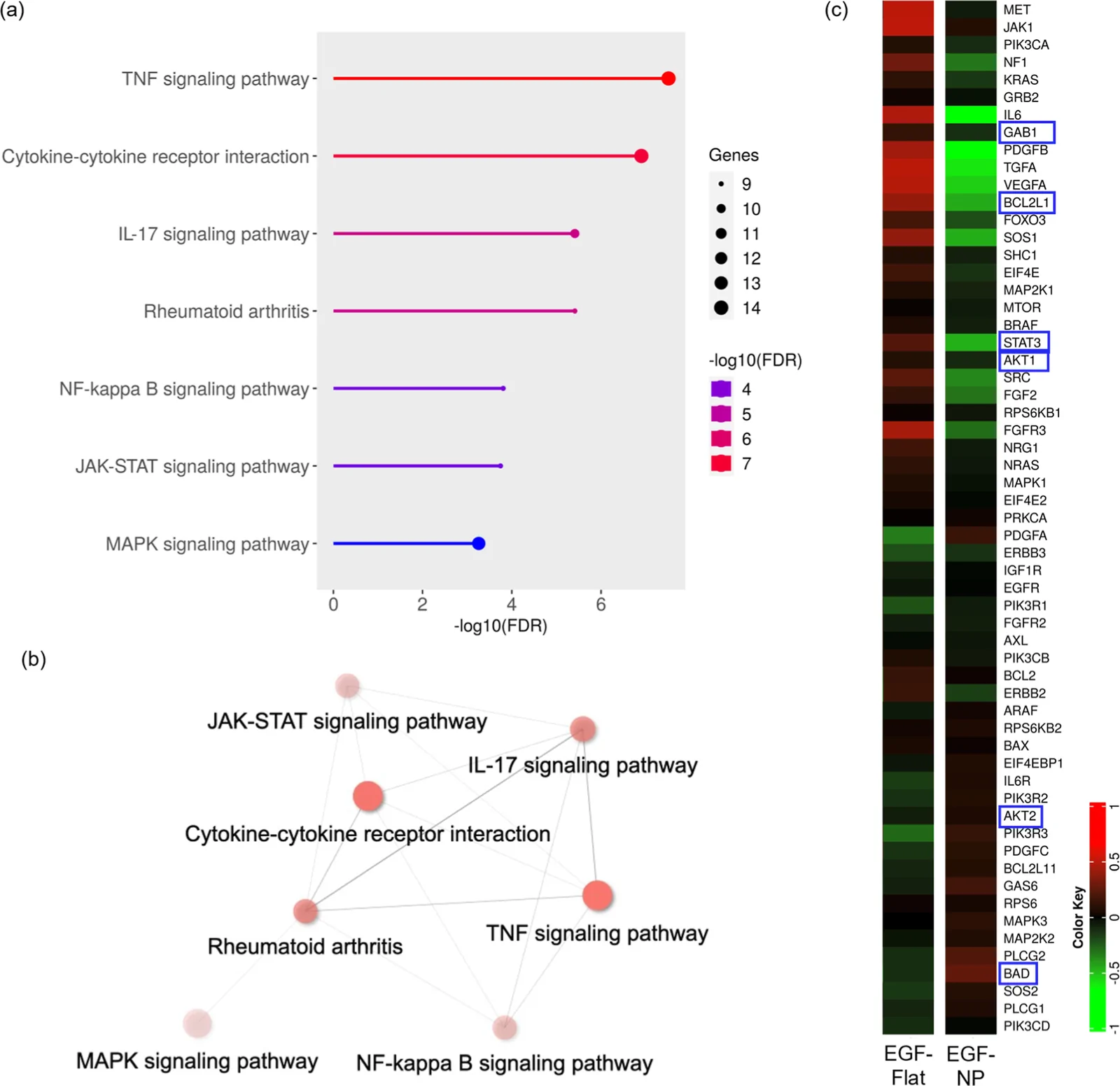
Transcriptomic profiling of cells treated with EGF-NP vs EGF-Flat. Total RNA was extracted from A431 cells after 4 h treatment with each material surface and subjected to RNA sequencing using the Illumina NextSeq 2000 platform. Differential gene expression was analyzed via the DRAGEN pipeline and BaseSpace, and visualized using iDEP. All data shown represent gene expression changes in EGF-NP-treated cells relative to EGF-Flat-treated cells. (a) KEGG enrichment plot generated from iDEP Bicluster analysis, showing significantly downregulated pathways in cells treated with EGF-NP compared to EGF-Flat. (b) KEGG pathway network analysis highlighting clusters of suppressed signaling pathways. (c) Heatmap comparing gene expression profiles between EGF-NP and EGF-Flat. Genes were selected based on the downregulated pathways identified in (a). RNA-seq analysis was performed using data obtained from three independent biological experiments.

### 2.4 Apoptosis Induction Requires EGFR Stimulation via Nanocurved Surface

To investigate the essential features of apoptosis induction by the nanocurved EGF-installed materials, we transitioned from surface-immobilized nanoparticles to SiNWs, which enable the precise control of particle spacing and ligand presentation at the nanoscale while achieving complete inhibition of endocytosis. SiNWs consist of nanoscale cylindrical structures whose length and intercylinder spacing can be controlled using nanoimprint lithography. Additionally, gold deposition on the SiNW surface enables thiol-gold chemistry for subsequent surface modification. Furthermore, because EGF is firmly immobilized on the SiNWs, endocytosis is completely blocked, preventing internalization of the EGFR–EGF complex. Nanostructures consisting of 200 nm gold nanoparticles arranged on top, with wires spaced at 400 nm intervals were fabricated (Figure 4a). Initially, the SiNWs were functionalized with PEG2k-silane on the silicon regions to prevent nonspecific adsorption of proteins in the culture solution. Subsequently, PEG5k-disulfide and DSU were modified on the gold regions, followed by reaction with EGF to prepare EGF-NW (Figure S3d, Supporting Information). Deformation of the cell membrane induced by the nanowires was visualized using confocal microscopy and focused ion beam system (FIB)–SEM (Figure 4b,c). Live-cell imaging was performed on cells with fluorescently stained membranes following the treatment with EGF-NW to observe membrane dynamics. Membrane perturbation was observed, manifesting as periodic dot-like structures aligned with the nanowire topography (Figure 4b). To further investigate this membrane deformation at the nanoscale, FIB-SEM analysis was also conducted. Given the technical limitations of direct SEM imaging of the apical cell surface, cancer cells were first allowed to adhere to fibronectin-coated SiNWs. Subsequently, a PEG-Flat substrate was applied to the apical surface of the cells to observe the approach of the nanowires toward the cell membrane. By creating cross sections using FIB, we directly visualized the interaction between the nanowires and the cell membrane. Importantly, FIB–SEM analysis revealed that the SiNWs directly penetrated the cell membrane (Figure 4c). Through these morphological observations, we successfully demonstrated that the EGF-NW physically drive cell membrane deformation. Additionally, to mimic the localized stimulation of EGF by nanowires, we prepared nanopatterned substrates by coating 200 nm thin-film gold regions on quartz glass. The gold regions were arrayed on the glass with a pitch of 500 nm (Figure 4d). This nanopatterned surface featured a thin gold film (∼5 nm), resulting in a structure that exerted negligible nanocurved material-mediated stimulation. The chemical functionality of the substrate was introduced in two steps. Initially, the glass regions were functionalized with PEG2k-triethoxysilane. Subsequently, the gold areas were modified by treatment with the *N*-succinimidyl ester-terminated PEG2k disulfide molecule, followed by the coupling of EGF to the tethered amines, resulting in the formation of an EGF nanodomain (EGF-ND) (Figure S3e, Supporting Information). To examine the ability of the EGF-ND to activate EGFR, we performed immunostaining for phosphorylated EGFR after applying the substrates to the apical side of the cells (Figure 4e). Confocal microscopy revealed that EGF-ND effectively activated EGFR at the cell membrane. Because the fluorescence signal was comparable to that of EGF-Flat, a substrate known to effectively activate EGFR, these results demonstrate that EGF-ND successfully induces robust receptor activation (Figure S8, Supporting Information). In contrast, the control PEG-Flat did not induce detectable phosphorylation of EGFR. We then evaluated the apoptosis-inducing ability of the EGF-SiNW and EGF-ND. EGF-NW, which provided both localized biochemical stimulation and nanocurved material-mediated stimulation, significantly activated caspase-3/7 (Figure 4f,h). In contrast, the EGF-ND, which offered only localized biochemical cues, failed to activate caspase-3/7 and showed an activity comparable to that of the PEG-Flat (Figure 4g,h). These results strongly suggest that the apoptotic activity of EGF nanostructures is attributable not only to localized stimulation by EGF but also to nanocurved material-mediated stimulation.

**Figure 4:**
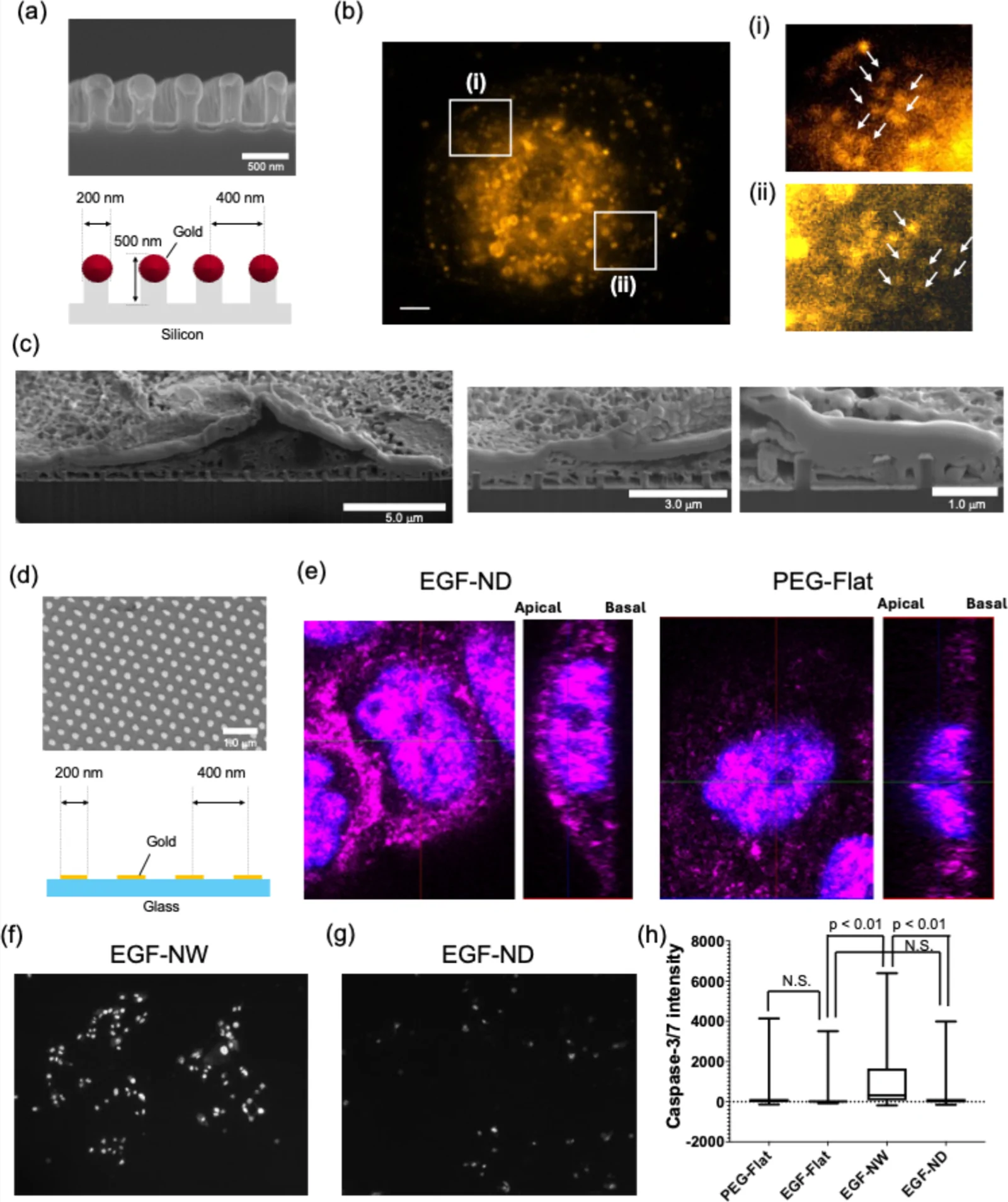
Induction of apoptosis by cooperative action of nanocurved material-mediated stimulation and localized EGF stimulation. (a) SEM image and material design of silicon nanowire. (b) Confocal fluorescence images of the apical cell membrane after addition with EGF-NW. The two right panels show magnified views of the regions indicated by the white squares in the main image. Arrows indicate the aligned dot-like membrane structures characteristic of the nanowire topography. Scale bar: 5 μm. (c) FIB–SEM image of A431 cells after treatment with EGF-NW. (d) SEM image and material design of nanopatterned surfaces. (e) Immunofluorescence staining of phosphorylated EGFR (pY1068; magenta) and nuclei (DAPI; blue) after a 4 h treatment with EGF-ND or PEG-Flat. Confocal images demonstrate robust EGFR activation on the plasma membrane of A431 cells exposed to EGF-ND, in contrast to the minimal signal observed with the PEG-Flat control. In the images, magenta indicates pEGFR and blue indicates DAPI. (f,g) Caspase-3/7 activity in A431 cells after 4 h treatment with (f) EGF-NW and (g) EGF-ND, detected by fluorescence microscopy. (h) Caspase-3/7 activity in A431 cells after 4 h treatment with PEG-Flat, EGF-Flat, EGF-NW and EGF-ND, detected by fluorescence microscopy. The data are shown as the mean ± SD from at least three independent experiments. Statistical differences were evaluated by Student’s *t*-test.

### 2.5 Apoptotic Activity is Mediated through PACSIN2-Dependent Signaling, a Membrane Curvature Sensor

Based on the results presented above, apoptosis induction by these nanostructures is attributable not only to localized EGF stimulation but also to nanocurved material-mediated stimulation of the cell membrane. Therefore, we directed our attention to the BAR domain, a well-characterized sensor of membrane curvature.^38^ The BAR domain plays a role in regulating cellular functions such as cytoskeletal organization and signal transduction by preferentially binding to curved regions of the cell membrane. Within the BAR protein family, PACSIN2 is involved in the membrane trafficking of EGF receptor and its downstream signal transduction.^39^

To examine the functional role of PACSIN2 in this process, we investigated the membrane localization of PACSIN2 after treatment with EGF-NP by immunofluorescence staining (Figure S9, Supporting Information). To accurately capture this dynamic recruitment, cells were stimulated with 20 nm EGF-NP, fixed with PFA, and the substrates were carefully removed prior to immunostaining. The distribution of PACSIN2 along the top–bottom axis was analyzed from confocal *z*-stacks (Figure S9a, Supporting Information). PACSIN2 fluorescence tended to accumulate toward the apical surface in EGF-NP-treated cells compared with control cells without substrate deposition (Figure S9b,c, Supporting Information). Next, using this approach, we calculated the proportion of PACSIN2 fluorescence intensity in the top 10% of the cell relative to the total cellular fluorescence intensity following treatment with PEG-Flat, EGF-Flat, and EGF-NP (Figure S9d, Supporting Information). EGF stimulation by EGF-Flat increased the fluorescence intensity of membrane-recruited PACSIN2 compared with PEG-Flat. However, EGF-NP induced significantly higher accumulation of PACSIN2 at the cell membrane than EGF-Flat. This clearly demonstrates that while EGF clustering alone recruits a baseline level of PACSIN2, nanocurved material-mediated stimulation further amplifies this recruitment.

We next examined the spatial relationship between PACSIN2 and phosphorylated EGFR (pEGFR Y1068) to address the link between nanostructure stimulation and signaling (Figure 5a). Colocalization analysis using Pearson’s correlation coefficient^40^ revealed that while EGF-Flat recruited PACSIN2, its colocalization with pEGFR remained moderate (average coefficient of ∼0.5) (Figures 5b and S10, Supporting Information). In contrast, the EGF-NP exhibited highly coordinated colocalization between PACSIN2 and pEGFR, with an average coefficient of ∼0.8. These findings strongly indicate that EGF-Flat merely recruits PACSIN2 to the membrane without efficient spatial coordination with active receptors. In contrast, the nanocurved material-mediated stimulation by EGF-NP actively organizes a localized signaling hub where PACSIN2 and pEGFR are tightly coupled. To further investigate EGFR dynamics, we examined the spatial relationship between phosphorylated EGFR and the early endosome marker Rab5 (Figure 5c). Colocalization analysis for both substrate conditions showed low correlation between pEGFR and Rab5 (average Pearson’s coefficient ∼ 0.28), comparable to that observed in EGF-Flat treated and control cells, indicating minimal EGFR internalization into early endosomes (Figures 5d and S11, Supporting Information). These results suggest that phosphorylated EGFR is not substantially internalized into early endosomes, but instead remains associated with the plasma membrane, where it colocalizes with PACSIN2. To determine whether PACSIN2 contributes to EGF-NP–induced apoptosis, we generated MDA-MB468 cells with PACSIN2 knockdown using the CRISPR-Cas9 system. Cas9 is an RNA-guided endonuclease derived from the bacterial adaptive immune system and widely adopted as a genome-editing tool.^41^ When combined with a guide RNA designed to recognize a specific DNA sequence, Cas9 introduces double-strand breaks at the target locus, thereby enabling precise gene disruption. A guide RNA specific to the PACSIN2 locus was introduced along with Cas9 into MDA-MB468 cells, leading to targeted cleavage of the gene. Western blotting confirmed that PACSIN2 expression was markedly reduced, nearly undetectable compared with empty vector-transfected control cells (Figure 5e). Next, we investigated the role of PACSIN2 in the apoptosis-inducing activity of EGF-NP using these cells (Figure 5f,g). When EGF-NP was applied to MDA-MB468 cells transfected with an empty vector, a marked increase in caspase-3/7 activity was observed. In contrast, PACSIN2-knockdown cells showed near-complete abolishment of caspase-3/7 activity upon EGF-NP treatment, with signal levels comparable to those observed with PEG-NP. Given that cells stimulated with localized EGF did not undergo apoptosis, this suggests that the apoptotic activity of the nanostructures requires nanocurved-mediated membrane deformation, transduced through PACSIN2. Furthermore, our findings reveal a cellular response mechanism, in which apoptosis is induced via EGFR signal transduction at the plasma membrane, independently of endocytosis, a process previously considered essential for EGFR-mediated apoptosis signaling and is governed by nanocurved-mediated stimulation of the cell membrane.

**Figure 5:**
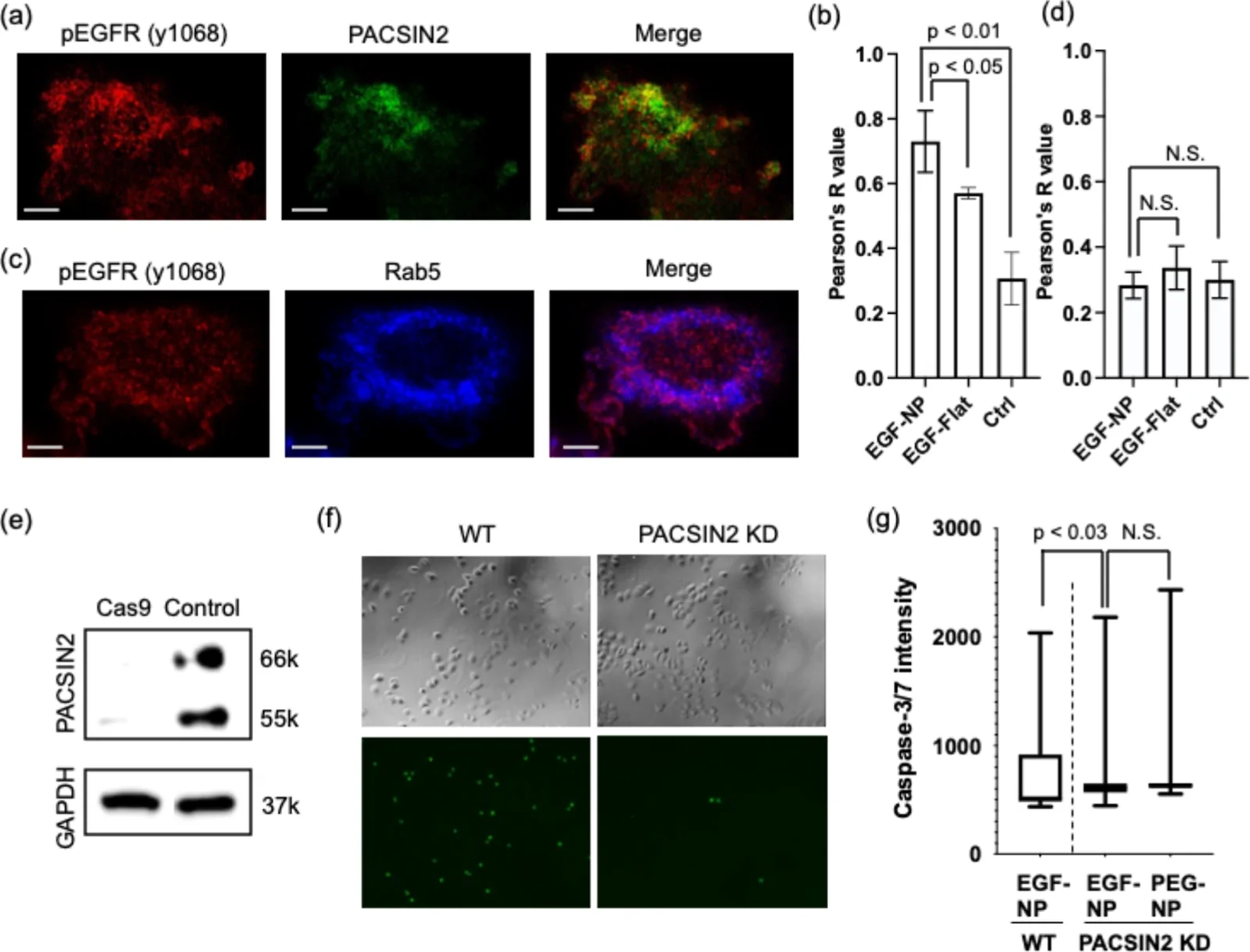
Colocalization of PACSIN2 with phosphorylated EGFR at the plasma membrane and its contribution to EGF-NP–induced apoptosis. (a) Confocal fluorescence imaging of PACSIN2 and pEGFR at the apical plasma membrane in cells treated with EGF-NP. Scale bars: 5 μm. (b) Pearson’s correlation coefficient–based colocalization analysis of PACSIN2–pEGFR for EGF-NP, EGF-Flat and control (ctrl). Ctrl refers to untreated cells without EGF-NP treatment. (c) Confocal fluorescence imaging of pEGFR and the early endosome marker Rab5 in the cytoplasmic plane. Scale bars: 5 μm. (d) Pearson’s correlation coefficient–based colocalization analysis of Rab5-pEGFR for EGF-NP, EGF-Flat and control (Ctrl). Ctrl refers to untreated cells without EGF-NP treatment. (e) Western blot analysis of PACSIN2 expression in MDA-MB468 cells transfected with Cas9 compared to control cells transfected with an empty vector. (f,g) Caspase-3/7 activity in PACSIN2-knockdown and wild-type MDA-MB468 cells after 4 h incubation with EGF-NP. The data are shown as the mean ± SD from at least three independent experiments. Statistical differences were evaluated by Student’s *t*-test.

## 3 Discussion

We propose a mechanistic model by which nanostructured EGF materials such as EGF-NP and EGF-NW acquire apoptosis-inducing activity (Figure 6). When applied to cancer cells, the nanocurved EGF materials induced nanoscale perturbation of the plasma membrane, leading to the localized activation of EGFR within these regions. The membrane curvature-sensing protein PACSIN2 is involved in signaling during this process, facilitating the detection of deformation signals and receptor signaling at the membrane interface. Our findings comprehensively demonstrate that neither structural alteration nor ligand presentation alone is sufficient to alter cell fate. Instead, the apoptotic response appears to be primarily mediated by the synergistic coordination of nanocurved material-mediated stimulation and biochemical EGF signaling, potentially accompanied by localized EGF clustering. In this synergistic process, the specific curvature profile of the nanostructure serves as a parameter determining the structural compatibility for PACSIN2 assembly, subsequently dictating the biological outcome. From a purely geometric perspective, smaller nanoparticles (such as 5 nm EGF-NP) possess a higher curvature. If the mechanism depended solely on the absolute degree of curvature, smaller nanoparticles should theoretically induce the strongest signaling responses. However, our results indicate that this biological response depends not on the mathematical curvature, but specifically on the structural compatibility between the induced membrane deformation and the curvature-sensing protein, PACSIN2. PACSIN2 is an F-BAR domain-containing protein critically involved in the formation of caveolae, which are specialized nanoscale membrane invaginations. Previous structural and biochemical studies have explicitly demonstrated that the F-BAR domain of PACSIN2 optimally induces and stabilizes membrane tubules with a diameter of approximately 50 nm.^42^ Furthermore, super-resolution microscopy analyses of intracellular PACSIN2 dynamics have revealed that it stably localizes to membrane structures with typical caveolae volumes (approximately 50–100 nm in diameter), whereas it fails to assemble on excessively small vesicles.^43^ Thus, for the extremely small 5 nm EGF-NP, the resulting membrane profile is simply too sharp to support the stable structural assembly of the PACSIN2 lattice, explaining their complete lack of apoptotic activity despite their high absolute curvature. Together, these structural perspectives elucidate that EGF-NP and EGF-NW exceeding 50 nm provide the optimal nanocurved material-mediated stimulation landscape required for stable PACSIN2 assembly, which subsequently shifts the EGF signaling cascade toward apoptosis.

**Figure 6:**
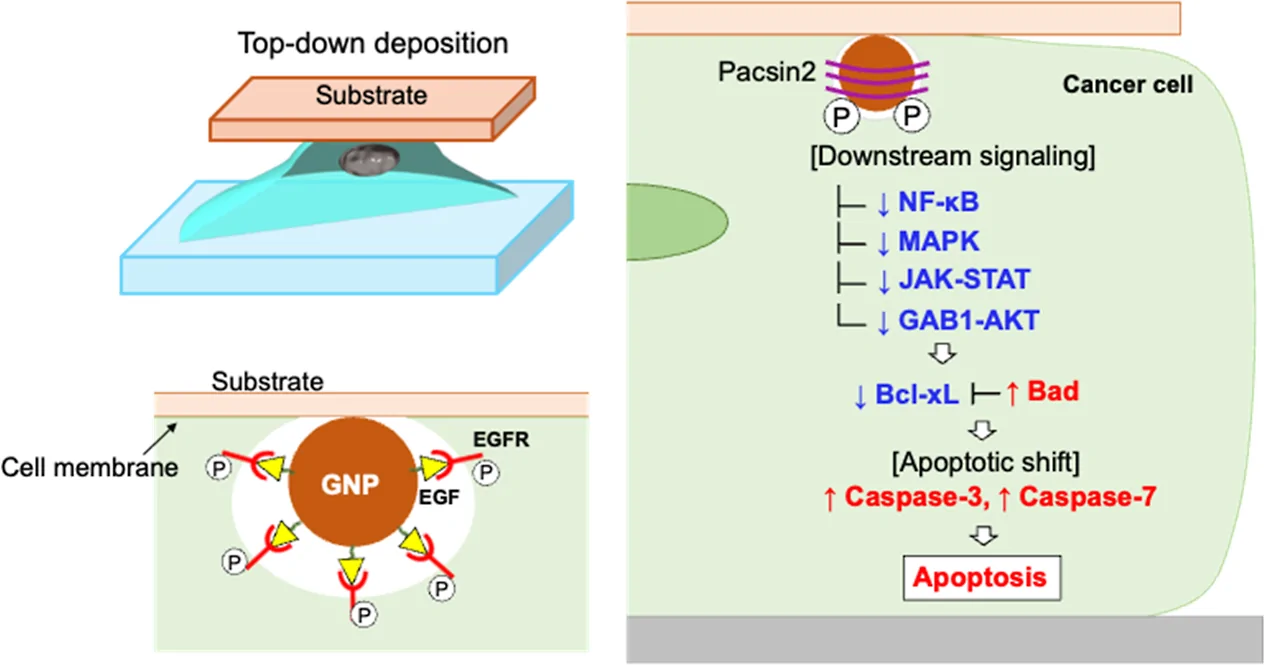
Expected mechanism by which EGF-NP acquires anticancer activity.

The enhanced biological efficacy of these nanocurved materials is fundamentally linked to the intensity of PACSIN2 mobilization at the plasma membrane. Confocal microscopic *Z*-stack quantitative analyses reveal that while conventional biochemical EGF stimulation via EGF-Flat substrates induces a baseline level of PACSIN2 membrane localization compared to untreated cells, the application of EGF-NP triggers a significantly more robust and pronounced accumulation of PACSIN2 at the membrane interface. This enhanced spatial recruitment is further corroborated by a remarkably stronger colocalization correlation between pEGFR and PACSIN2 on EGF-NP than on EGF-Flat surfaces. Nanocurved-driven PACSIN2 recruitment is a localized phenomenon distinct from conventional endocytic signaling pathways. On both EGF-NP and EGF-Flat substrates, pEGFR exhibits a complete absence of spatial colocalization with the early endosome marker Rab5, explicitly demonstrating that the pEGFR/PACSIN2 complexes remain retained at the cell surface interface rather than entering the intracellular endocytic machinery. Most importantly, our findings establish that the magnitude of this nanocurved material-mediated PACSIN2 mobilization directly determines downstream signaling strength and subsequent cell fate. While weaker PACSIN2 recruitment on EGF-Flat substrates allows the maintenance of high pro-survival signaling, the intensive and concentrated mobilization driven by EGF-NP successfully suppresses pERK activation, a pivotal downstream survival effector. Collectively, these results elucidate that the increased mobilization intensity of PACSIN2, uniquely afforded by nanocurved material-mediated stimulation, serves as the critical checkpoint that directly suppresses immediate survival cascades.

This localized signal reprogramming at the protein level is further substantiated by global transcriptomic profiling via RNA-seq. Analysis of the intracellular transcriptome revealed a unique response distinct from that triggered by flat EGF surfaces, characterized by a profound downregulation of multiple pro-survival signaling networks, including NF-κB, MAPK, and JAK-STAT. Alongside these changes, we observed decreased expression of the antiapoptotic gene Bcl-xL and upregulation of pro-apoptotic genes such as Bad. Although the PI3K/AKT pathway was not downregulated, the selective suppression of AKT1 expression may have contributed to the activation of pro-apoptotic transcriptional programs. These changes in gene expression collectively suggest a shift in intracellular signaling from survival to apoptosis. To definitively establish the causal requirement of this pathway, we utilized CRISPR-Cas9-mediated PACSIN2-knockdown cells. Upon treatment with EGF-NP, these cells completely failed to activate caspase-3 and caspase-7, indicating that PACSIN2 is not a mere secondary consequence but acts as a key player linking EGF nanostructure signaling to the apoptotic response. Previous studies have reported that treatment of cancer cells overexpressing EGFR with high concentrations of EGF induces prolonged accumulation of phosphorylated EGFR in early endosomes, leading to apoptotic activity.^44^ In contrast, our results suggest that the combination of nanocurved material-mediated stimulation and biochemical stimulation can induce apoptosis without requiring EGFR endocytosis. These findings highlight a mechanism by which EGF nanostructures reprogram cellular signaling and induce apoptosis in cancer cells.

However, it should be noted that direct visualization of the spatial colocalization of PACSIN2 assembling on individual EGF-nanostructures remains a technical challenge in our system. During sample preparation, particularly the intensive washing steps required for immunofluorescence, the detachment of the topographically delicate EGF-NP/NW detach from the apical membrane. Nevertheless, based on the macroscopic accumulation of PACSIN2 at nanocurved material-mediated stimulated regions and the complete loss of apoptotic activity in PACSIN2-knockdown cells, we reason that these nanostructures provide essential physical cues that drive this localized response. In biological systems, cells are located on extracellular matrices with nanoscale architectures, such as collagen and fibronectin fibrils, where growth factors may also be locally presented.^3^ Therefore, the nanocurved material-mediated stimulation observed in our system likely represents one of the factors that influence cancer cell fate in natural cellular microenvironments.

Recently, the interface between the cells and engineered materials has become an increasingly important focus in biomedical engineering.^45^ In particular, material-based patching strategies have been developed to enhance selectivity and increase local drug concentrations in specific cells or tissues.^46,47^ Biomaterials are primarily used as carriers to efficiently deliver anticancer agents. In contrast, our strategy uses EGF-functionalized nanostructures positioned at the subcellular level, enabling the material itself to function as an anticancer agent. This approach has the potential to minimize the systemic side effects and enable more precise and localized treatment. In future studies, these chimeric nanocurved EGF materials should be adapted to biocompatible soft materials, such as hydrogels or polymers, to explore their potential as a next-generation patch-based anticancer therapy.

## 4 Conclusion

In this study, nanocurved EGF materials induced apoptosis by exposing the apical sides of cancer cells to their surfaces. These nanostructures locally activated EGFR at the plasma membrane without inducing endocytosis and significantly enhanced caspase-3/7 activity compared with that on flat EGF-surfaces. Apoptosis was triggered only when EGFR stimulation was delivered through nanocurved materials that prompted the membrane mobilization of the curvature-sensing protein PACSIN2, operating in conjunction with EGF signaling. Neither stimulus alone was sufficient to induce caspase activation, highlighting the essential role of their synergistic effect. The size and spatial arrangement of the nanoparticles are also critical, because larger immobilized particles with nanoscale spacing significantly enhanced the apoptotic response. These findings indicate that PACSIN2 plays a key role in modulating EGFR signaling. Transcriptomic analysis supported this mechanism, showing the downregulation of pro-survival pathways and the upregulation of pro-apoptotic genes, indicating the reprogramming of intracellular signaling toward apoptosis. Furthermore, knockdown of PACSIN2 abolished apoptosis. Taken together, these findings support the conclusion that the membrane deformation generated by the nanocurved materials recruits PACSIN2, which in turn modulates the signaling cascade to induce apoptosis. Overall, our findings reveal a mechanism by which nanostructures can reshape growth factor signaling through coordinated nanocurved material-mediated and biochemical stimulation. Our strategy introduces a patch-based anticancer platform in which the material itself functions as a therapeutic agent, offering a promising alternative to conventional drug delivery systems by enabling localized treatment with reduced systemic side effects.

## 5 Methods

### 5.1 Reagents

Amine-poly­(ethylene glycol) (PEG) (*M*_w_ = 5000) was obtained from NOF corporation. Biotin-PEG-amine (*M*_w_ = 5000), biotin-PEG-thiol (*M*_w_ = 2000), silane-PEG-amine (*M*_w_ = 2000), mPEG-silane (*M*_w_ = 2000) and NHS-PEG-thiol (*M*_w_ = 2000) were purchased from Nanocs Inc. Epidermal growth factor (EGF) was obtained from Sigma-Aldrich. Dithiobis­(succinimidyl undecanoate) (DSU) was purchased from Dojindo. PEG-DSU was synthesized according to previously reported methods.

### 5.2 Synthesis of Disulfide with Biotinylated PEG-DSU (Biotin-PEG-DSU)

1:0.5:0.5 volume ratio of 5 mM biotin-PEG-amine (*M*_w_ = 5000), 5 mM DSU, and 10 mM triethylamine was dissolved in DMSO were mixed for 24 h at room temperature to obtain disulfide with biotinylated poly­(ethylene glycol). The solution was used for surface modification of the gold nanoparticles without purification.

### 5.3 Preparation of Biotinylated EGF-AuNPs

A total of 6 mL of gold nanoparticles (AuNPs, 20 nm diameter; BBI Solutions) was concentrated to 50 μL using a centrifugal concentrator (Vivaspin 20, MWCO 30,000; Sartorius) at 8000*g* for 30 min at room temperature. AuNPs were functionalized in a DMSO/water (9:1) solution containing 2 mM PEG-DSU, 0.5 mM biotin-PEG-DSU, and 0.5 mM DSU using a rotating mixer at room temperature for 16 h. The functionalized AuNPs were then washed with DMSO (23,000*g*, 60 min, three times) by centrifugation. In the final centrifugation step, AuNPs were resuspended in a DMSO/phosphate-buffered saline (PBS) (1:9) solution containing 6.1 μM EGF and 0.02% Tween-20. The mixture was gently shaken at 4 °C overnight. Finally, the AuNPs were washed with PBS containing 0.02% Tween-20 by centrifugation (18,000*g*, 30 min, 4 °C, six times) to obtain biotinylated EGF-AuNPs.

### 5.4 Preparation of EGF-NP

The gold substrates (5 × 5 or 16 × 16 mm) were ultrasonically cleaned in methanol for 5 min and dried under a stream of nitrogen. After cleaning the substrates using a UV–ozone cleaner (UV253, Filgen) for 20 min, they were immersed overnight at room temperature in a methanol solution of air-oxidized disulfide molecules bearing biotinylated PEG (*M*_w_ = 2,000, 1 mg/mL). After the reaction, the substrates were rinsed three times with methanol and dried under nitrogen to obtain biotinylated surfaces. Subsequently, 20 μg/mL of NeutrAvidin in PBST was applied to the substrates and incubated for 15 min at room temperature. After washing three times with PBST, 0.9 nM biotinylated EGF-AuNPs in PBST were added and allowed to react with NeutrAvidin for 30 min at room temperature. Finally, the substrates were washed three times with PBST to obtain EGF-NP.

### 5.5 Preparation of EGF-NW

Silicon-nanowires (SiNWs) were prepared by a regular nanoimprint lithography method. UV photoresist was spin-coated onto *n*-Si­(100) wafers and imprinted by a quartz mold to form a nanohole array. The imprinted resist is used to deposit MgO disks by an electron-gun evaporator onto the silicon. Bosch etching (cycling of 35 sccm SF_6_ and C_4_F_8_ gases) vertically removes silicon around the disks to form SiNWs. These NWs are cylindrically shaped, have straight-edged sides, flat tops, and are surrounded by a flat but roughened base substrate. The diameter and spacing of the SiNWs are controlled by the quartz mold and were ordinarily 200 nm in diameter and spaced 500 nm apart. The SiNW length is controlled by the plasma etching parameters where 3.5 min etching resulted in NWs 500 nm long. A gold film thickness equivalent to 100 nm when deposited on a planar perpendicular surface was deposited onto the SiNWs. The actual thickness of the Au film differs on the different parts of the SiNW substrate surface. The samples were then annealed in a hot-walled quartz tube furnace in an inert atmosphere of 50 sccm Argon at 300 Torr at 800 °C for 3 min. The SiNW substrates were sonicated in methanol for 5 min and dried under nitrogen air stream, cleaned with a UV–ozone cleaner with the O2-plasma for 5 min and placed in an UV–ozone cleaner for 1 h. The SiNWs were immersed in 0.3 mM mPEG-Silane (*M*_w_ = 2000, Nanocs) in methanol for 24 h at room temperature. After washing with methanol, and dried with nitrogen air stream, 8:2 volume ratio of 2.5 mM PEG5k-DSU and 2.5 mM DSU in DMSO were reacted with the gold areas for 24 h at room temperature, followed by washing with DMSO three times and drying with nitrogen. Finally, 4.5 μM EGF was reacted with the gold regions for 24 h at 4 °C. The SiNW substrates were washed with 3 times with PBST to obtain the EGF-NW.

### 5.6 Preparation of EGF-ND

Quartz glass substrates were pretreated by oxygen plasma ashing using a PB-600 system (Morita Engineering Co., Ltd.) under the following conditions: 300 W, 100 sccm O_2_ flow, for 3–5 min. Afterward, a dehydration bake was performed at 120 °C for 1 min or more. An electron-beam positive resist (ZEP520A, Zeon Corporation) was then spin-coated at 6000 rpm for 1 min to obtain a dry film thickness of approximately 100 nm, followed by baking on a hot plate at 180 °C for 5 min. Subsequently, an antistatic coating (Espacer 300Z, Showa Denko) was spin-coated at 2000 rpm for 30 s to form a ∼10 nm thick film. Electron-beam lithography was carried out at 50 kV using an ELS-7500EX system (Elionix Inc.). The antistatic layer was removed by rinsing with water, followed by nitrogen drying. Development was performed using n-amyl acetate for 1 min, followed by a 30 s rinse with ZMD-B, and dried under nitrogen. An additional oxygen plasma ashing step (300 W, 100 sccm O_2_ flow, 15–30 s) was then carried out. Metal deposition of Ti/Au (5/20 nm) was performed using an electron-beam evaporator (UEP-3000BS, ULVAC) under a working distance of 700 mm. Lift-off was conducted by soaking the samples in dimethylacetamide (DMA) at room temperature, followed by rinsing with isopropanol and nitrogen drying to obtain the gold nanodot array. The gold nanodot arrays were cleaned by washing five times with acetone, followed by 5 min ultrasonic cleaning in acetone. The same procedure was repeated with 2-propanol and hexane. After nitrogen drying, the substrates were cleaned using a UV–ozone cleaner for 1 h. The cleaned substrates were immersed in a 0.3 mM methoxy-PEG-silane (*M*_w_ = 2000) solution in methanol at room temperature for 24 h. After rinsing with methanol and drying under nitrogen, the substrates were further reacted with air-oxidized disulfide molecules bearing NHS-PEG (*M*_w_ = 2000, 1 mg/mL) in methanol for 24 h at room temperature, targeting the gold regions of the nanodot arrays. The substrates were then rinsed three times with methanol and dried under nitrogen. Finally, the substrates were incubated with 4.5 μM EGF at 4 °C for 24 h. After six washes with PBST, EGF-ND were obtained.

### 5.7 Preparation of EGF-Flat

The gold substrates (5 × 5 or 16 × 16 mm) were ultrasonically cleaned in methanol for 5 min and dried under a stream of nitrogen. After cleaning the substrates using a UV–ozone cleaner for 20 min, they were immersed overnight at room temperature in a methanol solution of disulfide molecules bearing NHS-PEG (*M*_w_ = 2000, 1 mg/mL). After the reaction, the substrates were rinsed three times with methanol and dried under nitrogen to obtain biotinylated surfaces. Subsequently, 4.5 μM EGF in PBST was applied to the substrates and incubated at 4 °C for 24 h. After six washes with PBST, EGF-Flat were obtained.

### 5.8 Dynamic Light Scattering Measurment

The diameters and size distributions of biotinylated EGF-AuNPs were measured by dynamic light scattering (DLS; Otsuka Electronics Co., Ltd.). The samples of biotinylated EGF-AuNPs were placed in disposable cuvettes, and measurements were performed at a scattering angle of 90° at room temperature.

Scanning electron microscope (SEM). Samples were mounted on aluminum stubs, coated with a 2 nm platinum layer using an E-1030 ion sputter coater (Hitachi, Tokyo, Japan), and examined with a scanning electron microscope (SU8000, Hitachi) under standard high-resolution imaging conditions at a scan rate of 5 kHz.

### 5.9 FIB–SEM

A fibronectin solution (6 μg/cm^2^) was added to a 3.5 cm dish, in which the SiNWs were immersed and incubated for 2 h at 37 °C. MDA-MB468 cells (1 × 10^5^ cells/well) were seeded onto the SiNWs and cultured for 24 h at 37 °C. After incubation, PEG-modified gold substrates were applied to the apical side of the cells for 4 h. The samples were subsequently fixed with 2.5% glutaraldehyde in PBS for 2 h at room temperature. Following fixation, the samples were rinsed with PBS, and the PEG-modified gold substrates were carefully removed. The samples were dehydrated by sequential washing with increasing concentrations of ethanol (10%, 25%, 40%, 50%, 70%, 80%, 90%, and 100%). Finally, the specimens were freeze-dried using a freeze-dryer (ES-2030, Hitachi). After freeze-drying, the samples were coated with a thin conductive layer using an osmium coater (HPC–1SW, Vacuum Device Inc., Japan). A plasma enhanced osmium coating was first applied to improve surface conductivity, followed by a platinum coating to prevent charging and beam-induced damage during imaging. The coated samples were mounted on aluminum stubs and positioned on the specimen stage at a 57° tilt angle. The samples were examined using a focused ion beam/scanning electron microscope (FIB/SEM) system (Helios 650, FEI Company Japan Ltd.).

### 5.10 Cell Culture

All cell lines were obtained from the American Type Culture Collection (ATCC, Manassas, VA, USA) and RIKEN BRC (Ibaraki, Japan). A431 cells were maintained in Dulbecco’s modified Eagle medium (DMEM) supplemented with 10% heat-inactivated fetal bovine serum (FBS; Biowest, Nuallie, France), 100 U/mL penicillin, and 100 μg/mL streptomycin at 37 °C in a humidified atmosphere containing 5% CO_2_. MDA-MB468 cells (triple-negative breast cancer; ATCC, HTB-132) were cultured in DMEM supplemented with 10% heat-inactivated FBS, 0.2% MycoZap (Lonza), 100 U/mL penicillin, and 100 μg/mL streptomycin under the same incubation conditions (37 °C, 5% CO_2_). Because this study used only established, commercially available cell lines and did not involve human participants, human-derived primary samples, or live animals, institutional ethics approval was not required.

### 5.11 Western-Blotting

A431 cells were seeded in 6 cm dishes at a density of 1.0 × 10^6^ cells per dish and cultured for 24 h at 37 °C. Cells were then serum-starved for 4 h at 37 °C prior to stimulation. Serum-starved cells were treated with EGF-AuNP-surfaces or EGF-surfaces for 15, 30, 60 min at 37 °C. After treatment, cells were washed twice with ice-cold PBS and lysed in radioimmunoprecipitation assay (RIPA) buffer (Nacalai Tesque) for 30 min at 4 °C. The resulting cell suspension was scraped, collected, and centrifuged at 13,000*g* for 10 min at 4 °C. Total protein concentration in the supernatant was quantified using the DC protein assay (Bio-Rad), with BSA as the standard. The lysates were mixed with SDS sample buffer containing 20 mM 2-mercaptoethanol (Wako), and boiled at 95 °C for 5 min. Proteins were separated by SDS-PAGE using a 10% Mini-PROTEAN TGX Gel (Bio-Rad) and transferred to a nitrocellulose membrane (Bio-Rad) at 20 V for 1 h. Membranes were blocked in 5% BSA in TBST (tris-buffered saline with 0.1% Tween-20) for 1 h at room temperature and incubated overnight at 4 °C with the following primary antibodies: anti-pEGFR (phospho Y1068, *M*_w_ ≈ 170 kDa, 1:1000, Abcam, catalogue number: ab32430, lot number: GR127111–39), anti-pERK (M_w_ ≈ 40 kDa, 1:1000, Cell Signaling Technology, catalogue number: 4370S, lot number: 24), anti-PACSIN2 (*M*_w_ ≈ 60 kDa, 1:500, Proteintech, catalogue number: PTG10518–2-AP, lot number: not provide), and anti-GAPDH (*M*_w_ ≈ 36 kDa, 1:2000, Abcam, catalogue number: ab181603, lot number: GR200347–34). After washing with TBST, membranes were incubated with HRP-conjugated secondary antibodies. Signals were visualized using the SuperSignal West Dura chemiluminescent substrate (Thermo Scientific) and imaged using a C–DiGit blot scanner with Image Studio software (LI-COR, USA).

### 5.12 Caspase-3/7 Detection

A431 cells and MDA-MB468 cells were seeded in 48-well plates at a density of 4.0 × 10^4^ cells per well and incubated at 37 °C for 24 h. The cells were then serum-starved for 4 h at 37 °C. After starvation, each sample were gently applied to the cells and incubated at 37 °C for 4 h. Following incubation, the cells were washed with Hank’s balanced salt solution (HBSS) containing 5% FBS and then treated with 5 μM CellEvent Caspase-3/7 Green Detection Reagent (Thermo Fisher Scientific) for 30 min at 37 °C. Fluorescence images were acquired using an Axiovert 200 microscope (Zeiss) equipped with a mercury arc lamp. All image acquisition systems were controlled using MetaMorph software (Molecular Devices), and the captured images were processed using ImageJ. To ensure the reliability and consistency of each independent experimental run, PEG-flat was consistently included as an internal negative control. Experimental batches were strictly validated and proceeded to image analysis only when this internal negative control confirmed the absence of nonspecific background signals and unexpected cell death.

### 5.13 RNA Isolation and RNA Sequencing

RNA was collected at the indicated time points using the RNeasy MicroKit (QIAGEN, 74004) according to the manufacturer’s protocol. A431 cells were seeded at a density of 1.0 × 10^6^ cells on 16 × 16 mm cover glasses placed in 6-well plates and cultured for 24 h at 37 °C. Cells were then serum-starved for 4 h at 37 °C prior to stimulation. Serum-starved cells were treated with EGF-AuNP-surfaces or EGF-surfaces (16 × 16 mm) for 4 h at 37 °C. At this stage, each surface was applied in alignment with the cover glass to ensure proper contact during incubation. After treatment, EGF-AuNP-surfaces or EGF-surfaces were removed, and only the cover glasses with attached cells were transferred to new 6-well plates and washed with PBS. Total RNA was extracted at designated time points using the RNeasy mini kit (QIAGEN) according to the manufacturer’s instructions. Approximately 500 ng of RNA was used for sequencing. The library preparation and indexing was conducted using Illumina stranded mRNA Prep and IlluminaR RNA UD indexes. Quality and library size was measured by E-gel power snap system (Thermo). RNA sequencing was performed by illumina NextSeq2000 with the DRAGEN Bio-IT Platform using NextSeq1000/2000 P2 reagents (100 cycles). FASTQ files were automatically generated through onboard DRAGEN pipeline processing. Differential expression analysis was conducted using Illumina BaseSpace sequence Hub’s RNA-Seq analysis App, which includes quality control, transcript quantification, and statistical analysis. The resulting CSV files containing differentially expressed genes were subsequently uploaded to iDEP2.0 for downstream bioinformatics analyses, including pathway analysis, hierarchical clustering and heatmap visualization.

### 5.14 Immunostaining

The cells were fixed with 4% paraformaldehyde for 15 min, rinsed with PBS, and quenched with 5% glycine. They were then permeabilized using 0.5% Triton X-100 in PBS for 5 min at room temperature, followed by PBS washes. Fixed cells were blocked with 5% bovine serum albumin (BSA) for 1 h at room temperature and incubated overnight at 4 °C with rabbit anti-EGFR (phospho Y1068) (1:1000 dilution, abcam), mouse anti-EGFR (phospho Y1068) (1:100 dilution, Cell Signaling Technology), rabbit anti-PACSIN2 (1:100 dilution, Proteintech), rabbit anti-Rab5 (1:1000 dilution, abcam). The next day, samples were washed three times with PBS and incubated with Alexa Fluor 488 goat antirabbit antibody (1:1000, ThermoFisher Scientific), Alexa Fluor 546 goat antimouse antibody, Alexa Fluor 568-conjugated chicken antirabbit antibody (1:1000, ThermoFisher Scientific) and Hoechst 33342 (1:1000, Thermo Fisher Scientific). Finally, the samples were washed three times with PBS and immediately imaged. When necessary, the samples were mounted in 50% glycerol to prevent evaporation during fluorescence imaging. Fluorescence images were acquired using a confocal laser scanning microscope (LSM710 or LSM 900, Zeiss) equipped with a 63× oil-immersion objective.

### 5.15 PACSIN2 Knock-Down by CRISPR-Cas9

PACSIN2 knockdown in MDA-MB468 cells was carried out using the CRISPR-Cas9 system. cDNAs for the N- and C-terminal fragments of codon-optimized *Streptococcus pyogenes* Cas9, both carrying an SV40 nuclear localization signal, were amplified from Addgene plasmid 42230. A plasmid encoding Cas9 was cotransfected with a guide RNA (gRNA) targeting the PACSIN2 gene. The gRNA sequence used was 5′-ACTACAAGCGGACTGTGAAG-3′, selected based on predicted high on–target activity and minimal off-target effects. Plasmid DNA was introduced into cells by electroporation using a 4D-Nucleofector system (Lonza), following the manufacturer’s instructions. After electroporation, cells were incubated under standard conditions and harvested 48 h later. Knockdown efficiency was evaluated by Western blotting using an anti-PACSIN2 antibody.

### 5.16 Statistical Analysis

The statistical analyses for the chosen two groups were performed using Student’s *t*-test for the technical replicates indicated in each figure. All the error bars represent standard deviations.

## Supplementary Material



## Notes

Detailed preparation methods for various EGF-functionalized surfaces; dynamic light scattering characterization; apoptosis-inducing activity assays; phase-contrast images; and confocal immunofluorescence images of pEGFR, PACSIN2, and Rab5 distributions (Figures S1–S11) (PDF)

## References

[1] Levental I., Lyman E. (2023). Regulation of membrane protein structure and function by their lipid
nano-environment. Nat. Rev. Mol. Cell Biol..

[2] van
Deventer S., Arp A. B., van Spriel A. B. (2021). Dynamic
Plasma Membrane Organization: A Complex Symphony. Trends Cell Biol..

[3] Karamanos N. K., Theocharis A. D., Piperigkou Z., Manou D., Passi A., Skandalis S. S., Vynios D. H., Orian-Rousseau V., Ricard-Blum S., Schmelzer C. E. H. (2021). A guide to the composition
and functions of the extracellular matrix. FEBS
J..

[4] Zalba S., ten Hagen T. L. M. (2017). Cell
membrane modulation as adjuvant in cancer therapy. Cancer Treat. Rev..

[5] Chen Z., Lin Y.-T., Salehi H., Che Z., Zhu Y., Ding J., Sheng B., Zhu R., Jiao P. (2023). Advanced Fabrication
of Mechanical Metamaterials Based on Micro/Nanoscale Technology. Adv. Eng. Mater..

[6] Liu S., Hoess P., Ries J. (2022). Super-Resolution Microscopy for Structural
Cell Biology. Annu. Rev. Biophys..

[7] Zhang A., Fang J., Li X., Wang J., Chen M., Chen H.-j., He G., Xie X. (2022). Cellular nanointerface
of vertical nanostructure arrays and its applications. Nanoscale Adv..

[8] Fischer R. S., Sun X., Baird M. A., Hourwitz M. J., Seo B. R., Pasapera A. M., Mehta S. B., Losert W., Fischbach C., Fourkas J. T. (2021). Contractility, focal adhesion orientation,
and stress fiber orientation drive cancer cell polarity and migration
along wavy ECM substrates. Proc. Natl. Acad.
Sci. U. S. A..

[9] Arnold M., Schwieder M., Blümmel J., Cavalcanti-Adam E. A., López-Garcia M., Kessler H., Geiger B., Spatz J. P. (2009). Cell interactions
with hierarchically structured nano-patterned adhesive surfaces. Soft Matter.

[10] Oria R., Wiegand T., Escribano J., Elosegui-Artola A., Uriarte J. J., Moreno-Pulido C., Platzman I., Delcanale P., Albertazzi L., Navajas D. (2017). Force loading explains
spatial sensing of ligands by cells. Nature.

[11] Zhao W., Hanson L., Lou H.-Y., Akamatsu M., Chowdary P. D., Santoro F., Marks J. R., Grassart A., Drubin D. G., Cui Y. (2017). Nanoscale
manipulation of membrane curvature for probing
endocytosis in live cells. Nat. Nanotechnol..

[12] Zhang W., Lu C.-H., Nakamoto M. L., Tsai C.-T., Roy A. R., Lee C. E., Yang Y., Jahed Z., Li X., Cui B. (2023). Curved adhesions mediate
cell attachment to soft matrix fibres in
three dimensions. Nat. Cell Biol..

[13] Yamamoto S., Chang C.-J., Kamimura M., Nakanishi J. (2025). Exploring
anti-cancer activities of epidermal growth factor-immobilized polymeric
nanoparticles. Sci. Technol. Adv. Mater..

[14] Yamamoto S., Nakanishi J. (2021). Epidermal
Growth Factor-gold Nanoparticle Conjugates-induced
Cellular Responses: Effect of Interfacial Parameters between Cell
and Nanoparticle. Anal. Sci..

[15] Yamamoto S., Iwamaru Y., Shimizu Y., Ueda Y., Sato M., Yamaguchi K., Nakanishi J. (2019). Epidermal
growth factor-nanoparticle
conjugates change the activity from anti-apoptotic to pro-apoptotic
at membrane rafts. Acta Biomater..

[16] Ito Y. (2008). Covalently
immobilized biosignal molecule materials for tissue engineering. Soft Matter.

[17] Wu L.-C., Tada S., Isoshima T., Serizawa T., Ito Y. (2023). Photo-reactive
polymers for the immobilisation of epidermal growth factors. J. Mater. Chem. B.

[18] Yasami-Khiabani S., Karkhaneh A., Shokrgozar M. A., Amanzadeh A., Golkar M. (2020). Size effect of human epidermal growth factor-conjugated
polystyrene particles on cell proliferation. Biomater. Sci..

[19] Preta G., Cronin J. G., Sheldon I. M. (2015). Dynasore - not just a dynamin inhibitor. Cell Commun. Signaling.

[20] Wu L., Yu X., Feizpour A., Reinhard B. M. (2014). Nanoconjugation: a materials approach
to enhance epidermal growth factor induced apoptosis. Biomater. Sci..

[21] Wu L., Xu F., Reinhard B. M. (2016). Nanoconjugation prolongs endosomal signaling of the
epidermal growth factor receptor and enhances apoptosis. Nanoscale.

[22] Ito Y. (2019). Growth Factor
Engineering for Biomaterials. ACS Biomater.
Sci. Eng..

[23] Yamamoto S., Nakanishi J., Yamaguchi K. (2015). Development and Characterization
of Protein-gold-nanoparticle Conjugates bearing Photocleavable Polymers. J. Photopolym. Sci. Technol..

[24] Roskoski R. (2012). ERK1/2 MAP
kinases: Structure, function, and regulation. Pharmacol. Res..

[25] Sahoo G., Samal D., Khandayataray P., Murthy M. K. (2023). A Review on Caspases:
Key Regulators of Biological Activities and Apoptosis. Mol. Neurobiol..

[26] Valon L., Levayer R. (2019). Dying under pressure: cellular characterisation
and
in vivo functions of cell death induced by compaction. Biol. Cell.

[27] Coyer S. R., Singh A., Dumbauld D. W., Calderwood D. A., Craig S. W., Delamarche E., García A. J. (2012). Nanopatterning
reveals an ECM area threshold for focal adhesion assembly and force
transmission that is regulated by integrin activation and cytoskeleton
tension. J. Cell Sci..

[28] Gautrot J. E., Malmström J., Sundh M., Margadant C., Sonnenberg A., Sutherland D. S. (2014). The Nanoscale Geometrical Maturation
of Focal Adhesions Controls Stem Cell Differentiation and Mechanotransduction. Nano Lett..

[29] Zhang W., Liu H. T. (2002). MAPK signal pathways in the regulation of cell proliferation
in mammalian cells. Cell Res..

[30] Guo Q., Jin Y., Chen X., Ye X., Shen X., Lin M., Zeng C., Zhou T., Zhang J. (2024). NF-κB in biology
and targeted therapy: new insights and translational implications. Signal Transduction Targeted Ther..

[31] Xue C., Yao Q., Gu X., Shi Q., Yuan X., Chu Q., Bao Z., Lu J., Li L. (2023). Evolving cognition
of the JAK-STAT
signaling pathway: autoimmune disorders and cancer. Signal Transduction Targeted Ther..

[32] Jiang L., Luo M., Liu D., Chen B., Zhang W., Mai L., Zeng J., Huang N., Huang Y., Mo X. (2013). BAD overexpression inhibits
cell growth and induces apoptosis via
mitochondrial-dependent pathway in non-small cell lung cancer. Cancer Cell Int..

[33] Zhang X., Tang N., Hadden T. J., Rishi A. K. (2011). Akt, FoxO and regulation
of apoptosis. Biochim. Biophys. Acta Mol. Cell
Res..

[34] Aggarwal B. B. (2003). Signalling
pathways of the TNF superfamily: a double-edged sword. Nat. Rev. Immunol..

[35] Gaffen S. L. (2009). Erratum:
Structure and signalling in the IL-17 receptor family. Nat. Rev. Immunol..

[36] O’Shea J. J., Plenge R. (2012). JAK and STAT Signaling
Molecules in Immunoregulation
and Immune-Mediated Disease. Immunity.

[37] Greten F. R., Weber C. K., Greten T. F., Schneider G., Wagner M., Adler G., Schmid R. M. (2002). Stat3 and
NF-κB
activation prevents apoptosis in pancreatic carcinogenesis. Gastroenterology.

[38] Simunovic M., Evergren E., Callan-Jones A., Bassereau P. (2019). Curving Cells
Inside and Out: Roles of BAR Domain Proteins in Membrane Shaping and
Its Cellular Implications. Annu. Rev. Cell Dev.
Biol..

[39] de
Kreuk B.-J., Anthony E. C., Geerts D., Hordijk P. L. (2012). The F-BAR
Protein PACSIN2 Regulates Epidermal Growth Factor Receptor Internalization. J. Biol. Chem..

[40] Zhang A., Abdellatef S. A., Nakanishi J. (2023). Mechanistic investigation into selective
cytotoxic activities of gold nanoparticles functionalized with epidermal
growth factor variants. Anal. Sci..

[41] Pacesa M., Pelea O., Jinek M. (2024). Past, present,
and future of CRISPR
genome editing technologies. Cell.

[42] Senju Y., Itoh Y., Takano K., Hamada S., Suetsugu S. (2011). Essential
role of PACSIN2/syndapin-II in caveolae membrane sculpting. J. Cell Sci..

[43] Nishimura T., Suetsugu S. (2022). Super-resolution analysis of PACSIN2 and EHD2 at caveolae. PLoS One.

[44] Rush J. S., Quinalty L. M., Engelman L., Sherry D. M., Ceresa B. P. (2012). Endosomal
Accumulation of the Activated Epidermal Growth Factor Receptor (EGFR)
Induces Apoptosis. J. Biol. Chem..

[45] Hiwrale A., Bharati S., Pingale P., Rajput A. (2023). Nanofibers: A current
era in drug delivery system. Heliyon.

[46] Chen L., Nabil A., Fujisawa N., Oe E., Li K., Ebara M. (2023). A facile, flexible, and multifunctional
thermo-chemotherapy system
for customized treatment of drug-resistant breast cancer. J. Controlled Release.

[47] Omer A. M., Abd El-Monaem E. M., Eltaweil A. S., Tamer T. M., Mohy
Eldin M. S., Ouyang X.-k., Heydari A. (2024). Advances in stimuli-responsive
polymeric hydrogels for anticancer drug delivery: A review. J. Drug Delivery Sci. Technol..

