# Supplementary material for: Chimeric Nanocurved Materials Reprogram Receptor Signaling to Induce Apoptosis via a Curvature-Sensing Protein
Source: ACS Nano. 2026 Aug 5;20(33):23175–88. doi: 10.1021/acsnano.6c00463 (PMC13523741; doi:10.1021/acsnano.6c00463)
Supplement: Supplementary file 1 [file nn6c00463_si_001.pdf]

Supporting Information

Chimeric nanocurved materials reprogram receptor signaling to induce apoptosis via a curvature-sensing protein

Shota Yamamoto[†,*], *Naoki Fukata*[‡], *Wipakorn Jevasuwan*[‡], *Moritoshi Sato*[§,∥] *and Jun Nakanishi*[†,⊥,#,¶,*]

[†]Research Center for Macromolecules & Biomaterials, National Institute for Materials Science (NIMS), 1-1 Namiki, Tsukuba, Ibaraki 305-0044, Japan

[‡]Research Center for Materials Nanoarchitectonics, National Institute for Materials Science (NIMS), 1-1 Namiki, Tsukuba, Ibaraki 305-0044, Japan

[§]Graduate School of Arts and Sciences, The University of Tokyo, 3-8-1 Komaba, Meguro-ku, Tokyo 153-8902, Japan

[∥]Kanagawa Institute of Industrial Science and Technology, 3-2-1 Sakado, Takatsu-ku, Kawaski-shi, Kanagawa 213-0012, Japan

[⊥]Graduate School of Advanced Science and Engineering, Waseda University, 3-4-1 Okubo, Shinjuku-ku, Tokyo 169-8555, Japan

[#]Graduate School of Advanced Engineering, Tokyo University of Science, 6-3-1 Niijuku, Katsushika-ku, Tokyo 125-8585, Japan

[¶]Research Center for Autonomous Systems Materialogy (ASMat), Institute of Integrated Research (IIR), Institute of Science Tokyo (Science Tokyo), 4259 Nagatsuta-cho, Midori-ku, Yokohama, Kanagawa 226-8501, Japan

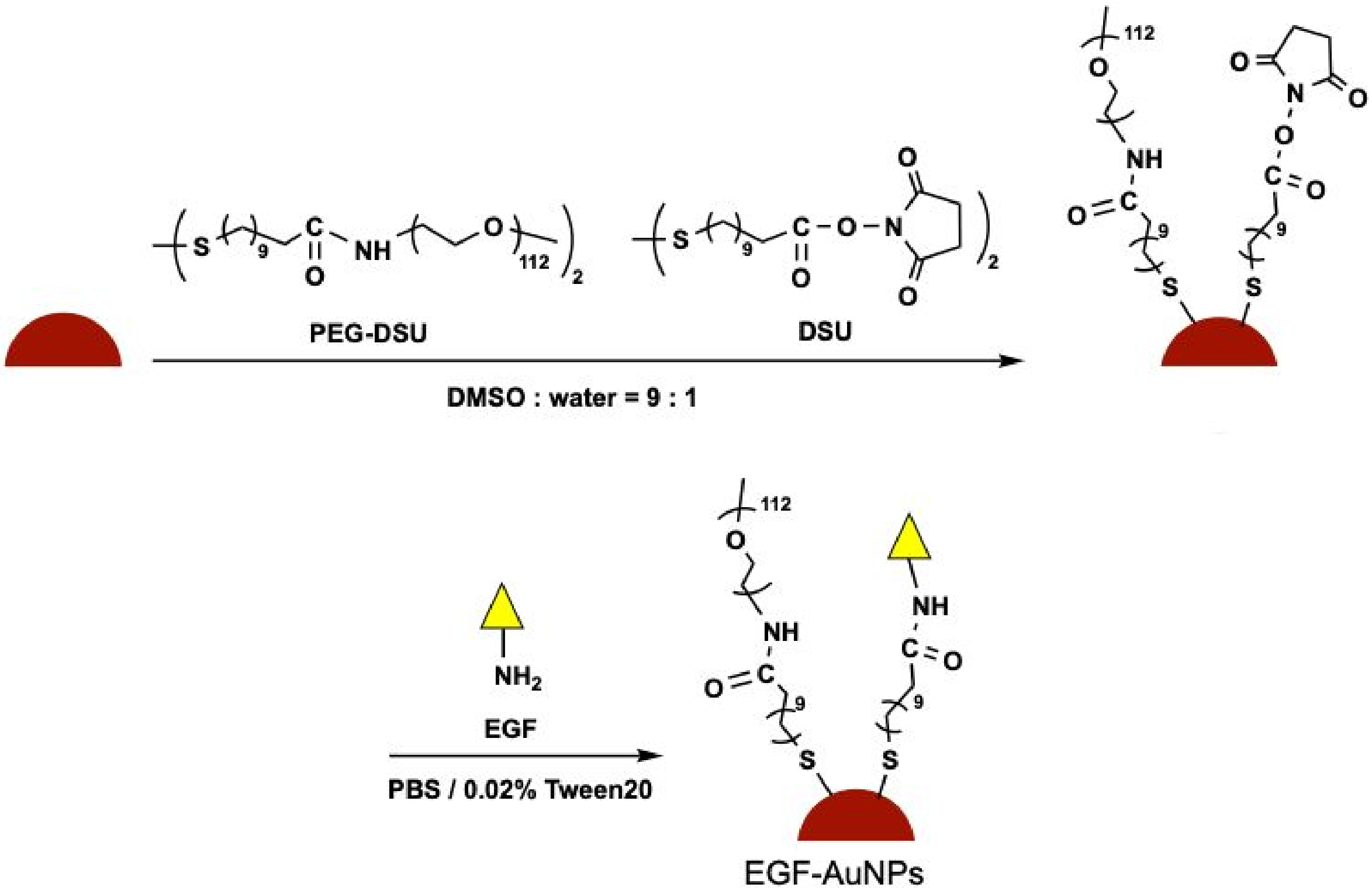

Figure S1. Preparation of EGF-AuNPs.

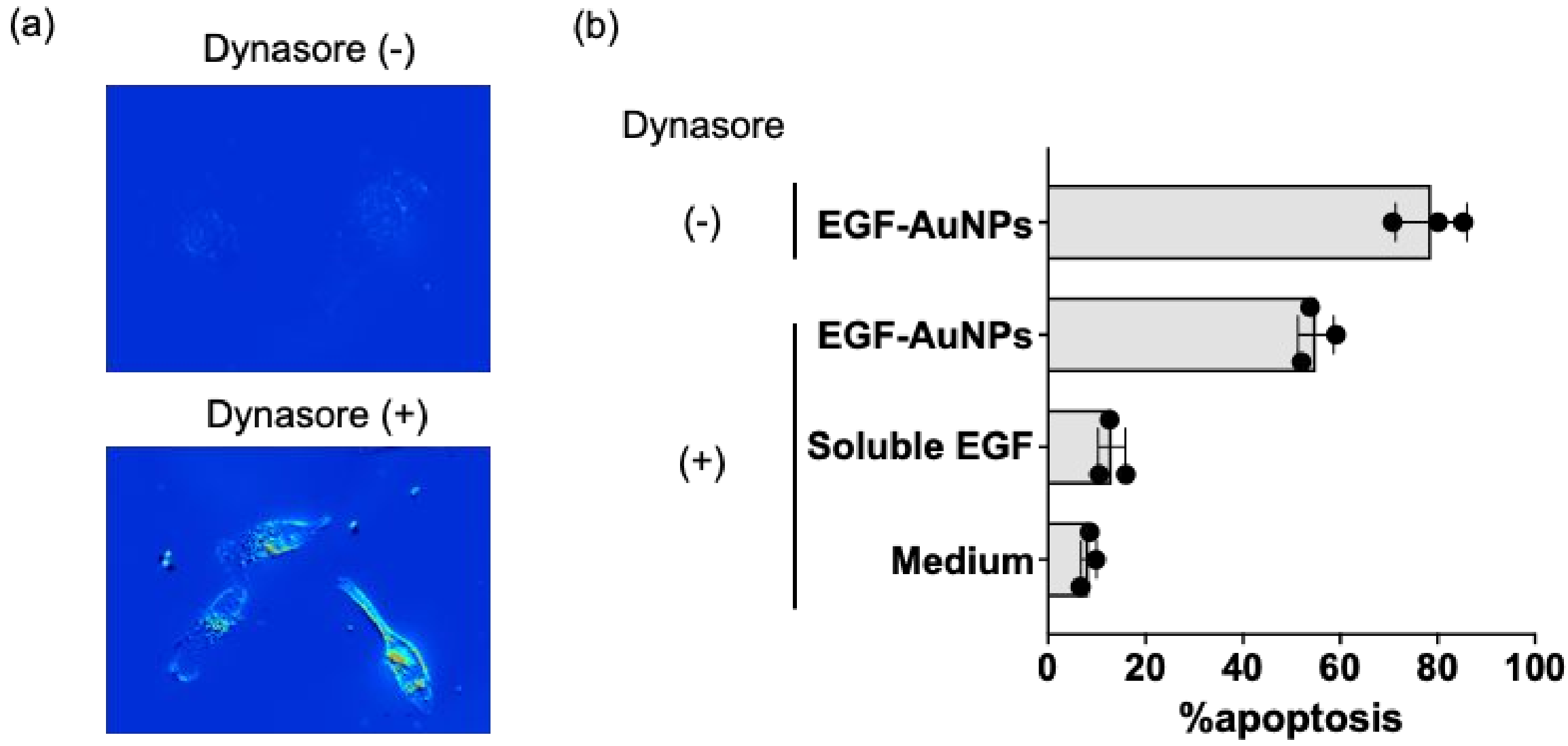

Figure S2. Apoptosis-inducing activity of EGF-AuNPs with or without dynasore. (a) Pseudo-colored ratiometric dark-field images of HeLa cells treated with 0.9 nM EGF-AuNPs for 4 hours. Images were generated by dividing the red-channel image (λ = 540 nm) by the green-channel image (λ = 480 nm). Warmer colors indicate regions where gold nanoparticles are present, due to enhanced red scattering by the particles. (b) Apoptotic activity of soluble EGF, EGF-auNPs, and medium against HeLa cells. The apoptotic cells was determined by Annexin V and propidium iodide (PI) staining after 72 hours. The apoptosis rate was determined based on the proportion of cells exhibiting fluorescence intensities greater than 3σ above the mean of untreated control cells. Data are presented as mean ± S.D. from three independent experiments. Statistical significance was evaluated using Student's t-test.

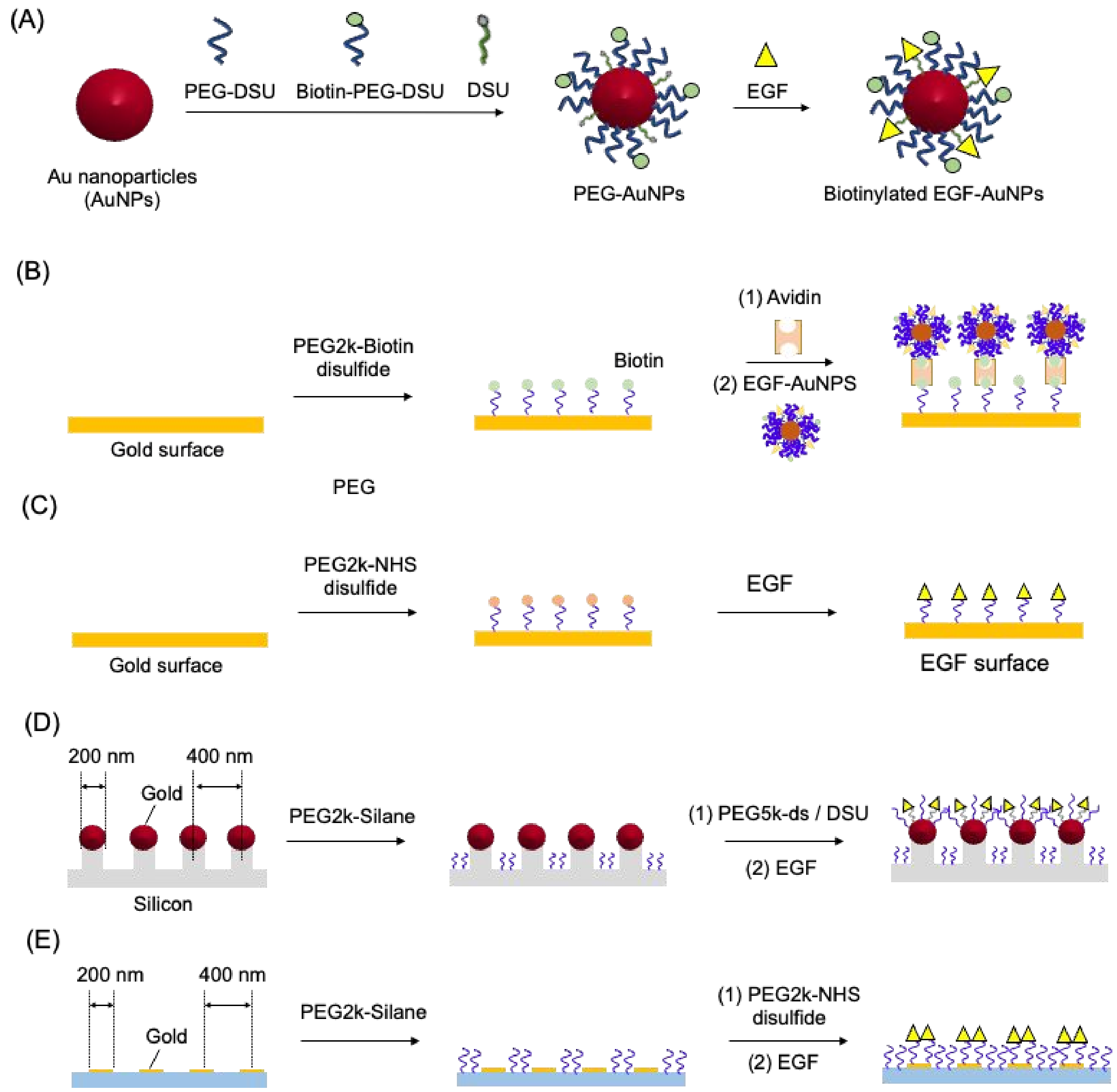

Figure S3. Preparation of (A) biotinylated EGF-AuNPs, (B) EGF-NP, (C) EGF-Flat, (D) EGF-NW and (E) EGF-ND.

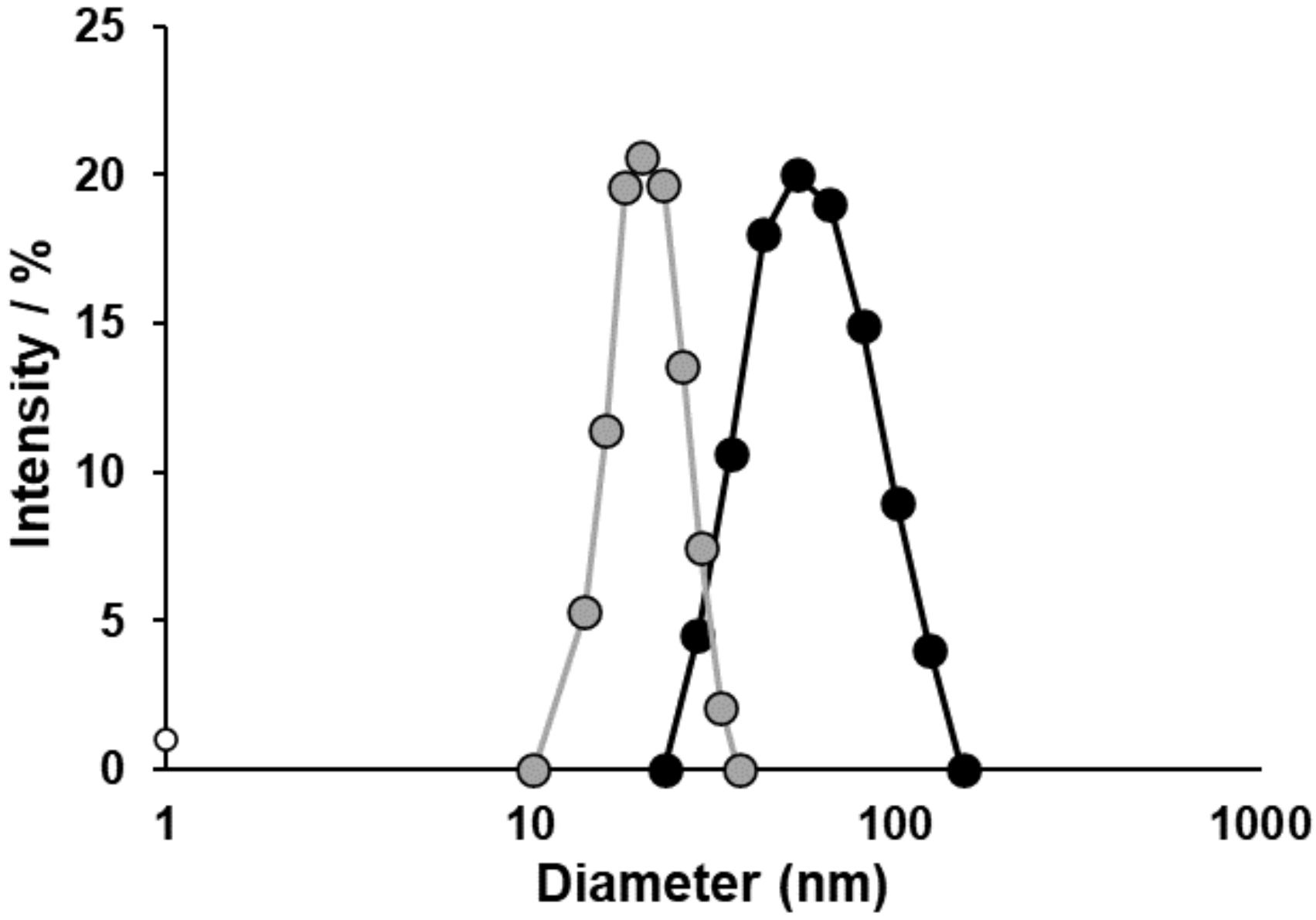

Figure S4. DLS study of the change in the particle distribution of GNPs (gray) and biotinylated EGF-AuNPs (black).

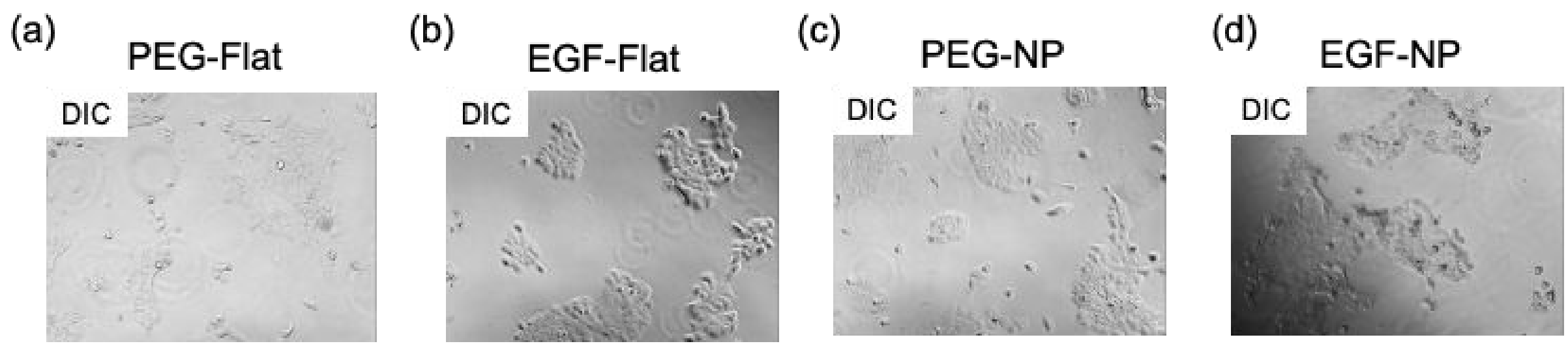

Figure S5. Phase-contrast images of A431 cells after 4-hour incubation with (a) PEG-Flat, (b) EGF-Flat, (c) PEG-NP and (d) EGF-NP.

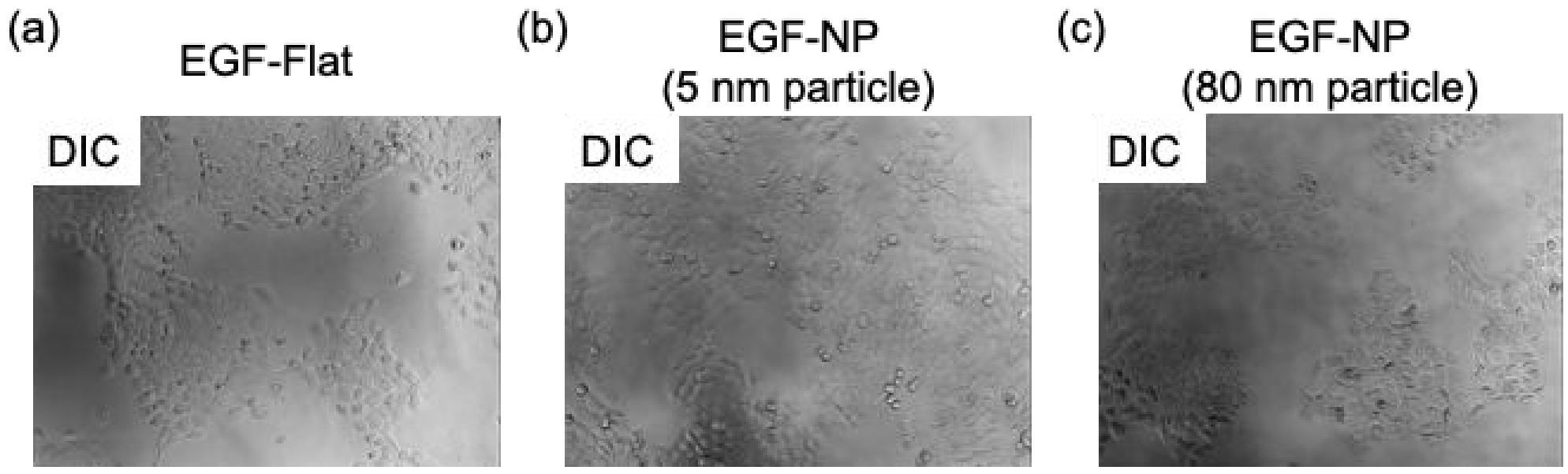

Figure S6. Phase-contrast images of A431 cells after 4-hour incubation with (a) EGF-Flat, (b) 5 nm EGF-NP, and (c) 80 nm EGF-NP.

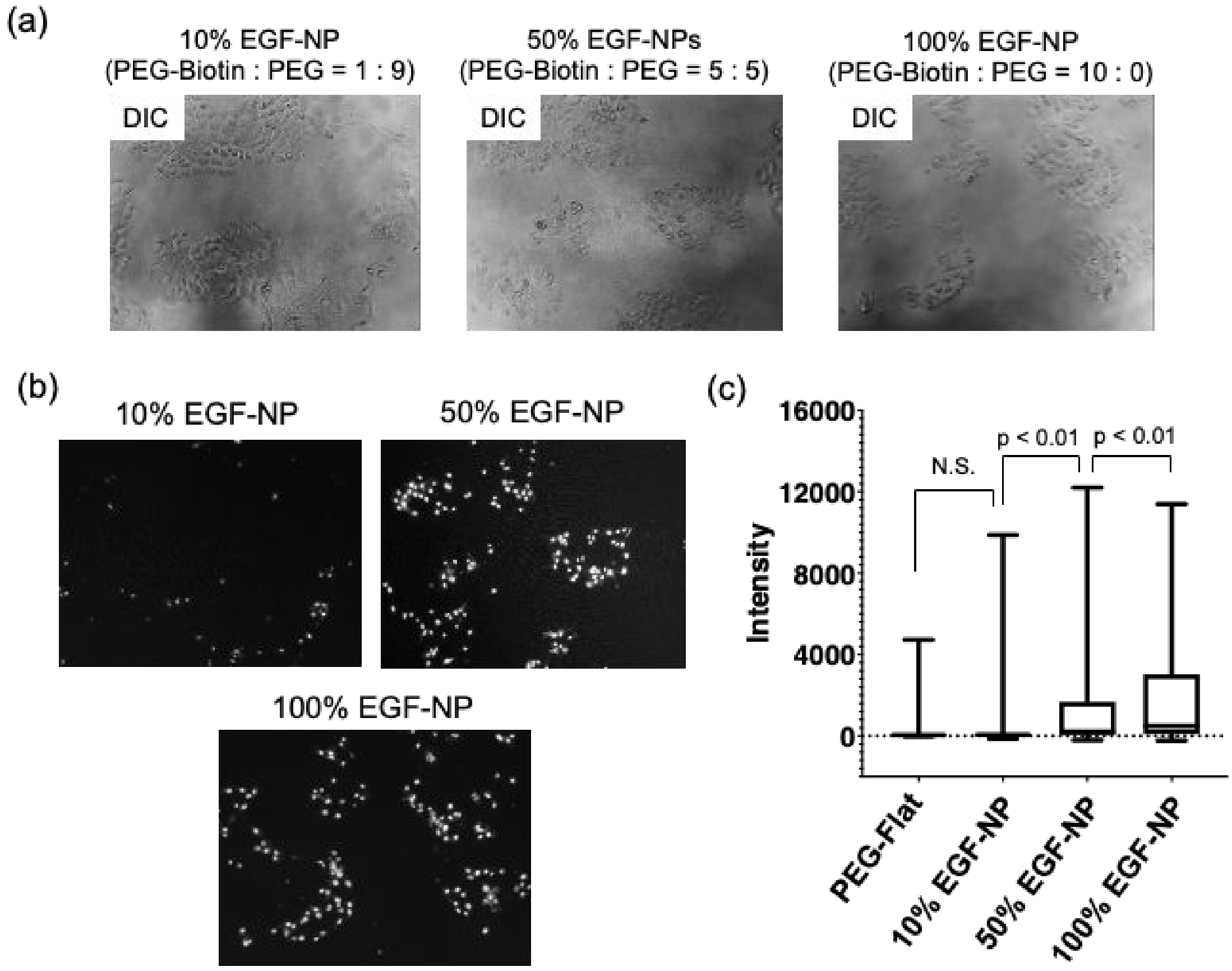

Figure S7. Impact of nanoparticle density on apoptosis induction. (a) Phase-contrast images of A431 cells after 4-hour incubation with 10%, 50% and 100% EGF-NP. (b) Fluorescence images showing caspase-3/7 activity in A431 cells after 4-hour incubation with 10%, 50%, and 100% EGF-NP. (c) Comparison of fluorescence intensities among PEG-Flat and EGF-NP. Data are presented as mean ± S.D. from three independent experiments. Statistical significance was evaluated using Student's t-test.

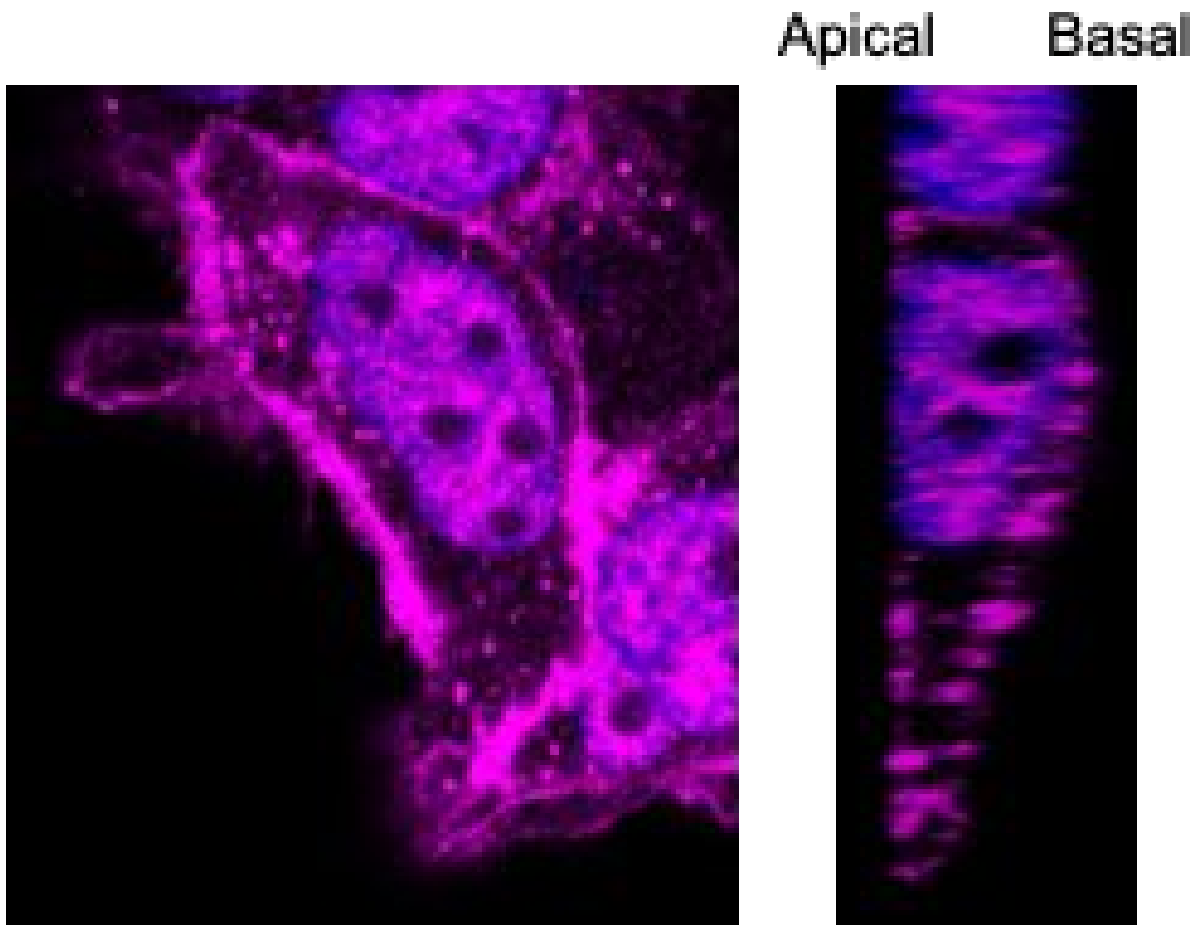

Figure S8. Immunofluorescence staining of phosphorylated EGFR (pY1068) and nuclei (DAPI) after a 4 h treatment with EGF-Flat. In the images, magenta indicates pEGFR and blue indicates DAPI.

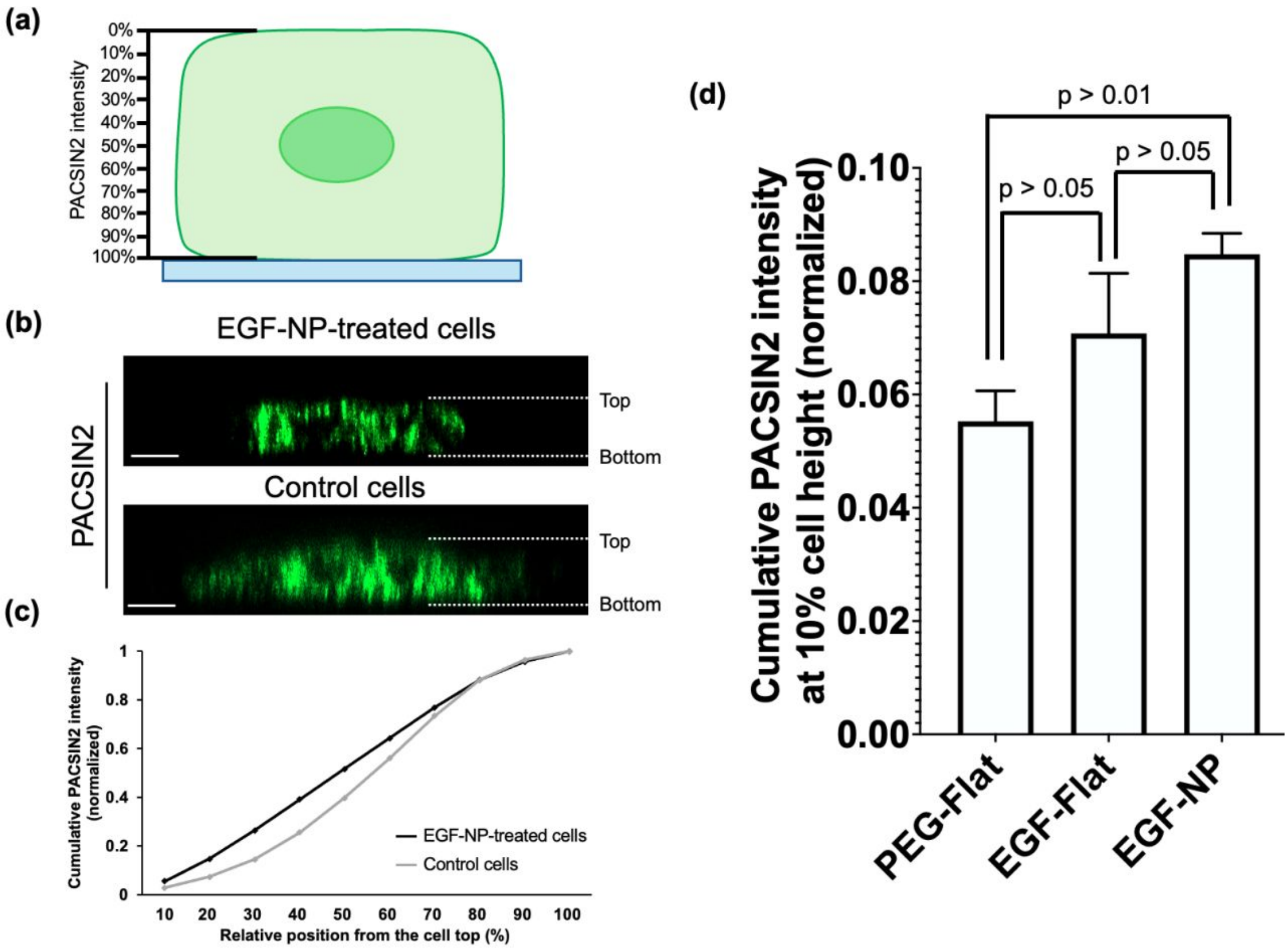

Figure S9. PACSIN2 distribution in cells. (a) Schematic illustration of the experimental procedure used to evaluate PACSIN2 accumulation at the apical membrane. (b) Orthogonal views of confocal z-stacks showing PACSIN2 fluorescence in EGF-NP-treated cells and control cells. Control cells refer to untreated cells without EGF-NP treatment. The cell top and bottom are indicated by white dashed lines. Scale bars: 5 µm. (c) Quantification of the cumulative PACSIN2 fluorescence intensity along the top–bottom axis. Confocal z-stacks consisted of 50 optical sections for EGF-NP-treated cells and 60 optical sections for control cells. To account for differences in cell height, the z-stacks were divided into ten equal segments from the cell top to the basal side. The fluorescence intensity in each segment was calculated as the sum of the intensities across 5 sections (EGF-NP-treated cells) or 6 sections (control cells). Cumulative PACSIN2 intensity was calculated from the cell top and normalized to the fluorescence integrated density of each cell. (d) Normalized cumulative PACSIN2 intensity within the top 10% of cell height after treatment with PEG-Flat, EGF-Flat, or EGF-NP. To minimize the effect of differences in cell morphology, cells in which PACSIN2 fluorescence could be observed across 60 optical sections from the cell top to the bottom were selected for analysis ($n \geq 4$ cells per condition). PACSIN2 fluorescence intensity within the top 10% of cell height was calculated from the cell top and normalized to the total PACSIN2 fluorescence intensity across the entire cell. Data are presented as mean ± S.D. from three independent experiments. Statistical significance was evaluated using Student's t-test.

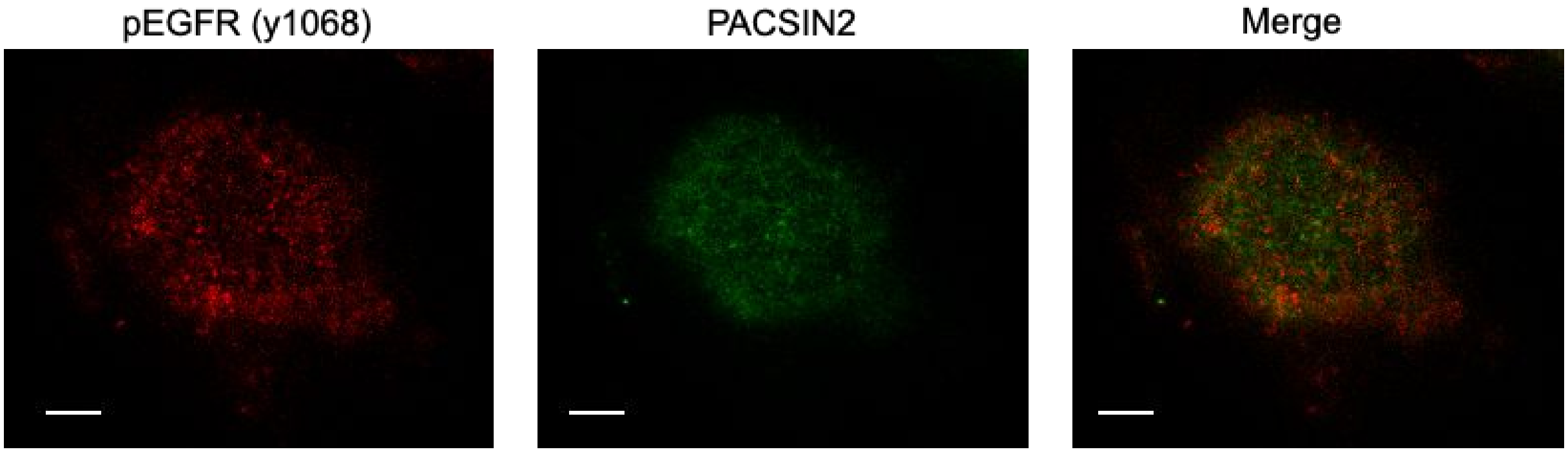

Figure S10. Confocal fluorescence imaging of PACSIN2 and pEGFR at the apical plasma membrane in cells treated with EGF-Flat. Scale bars: 5 μm.

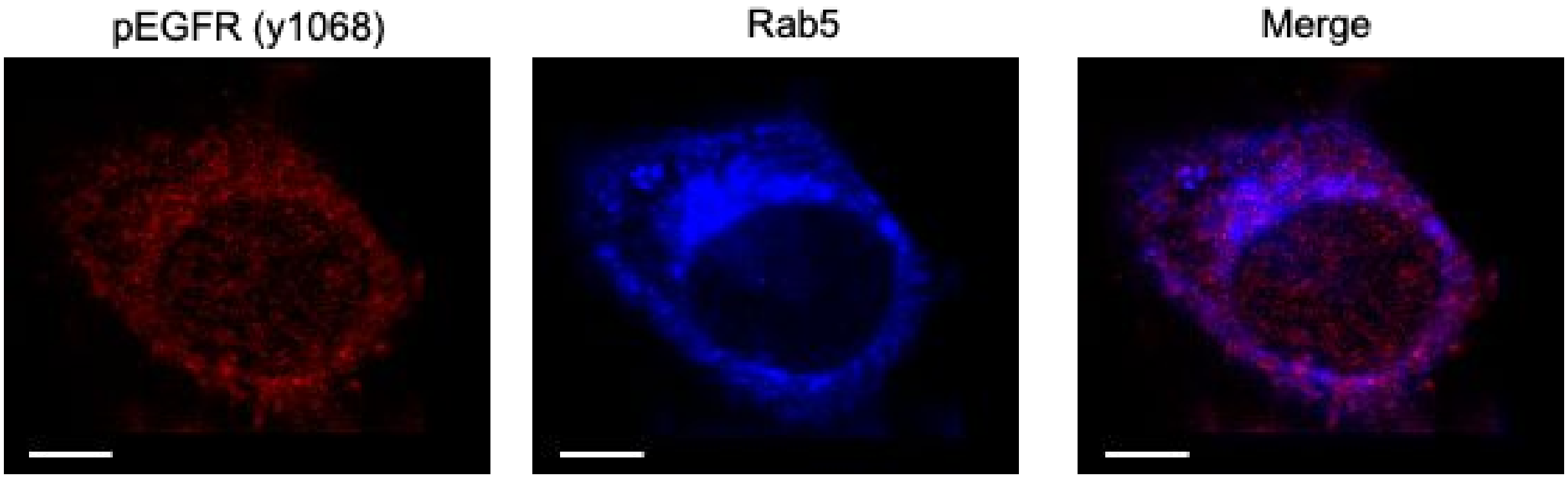

Figure S11. Confocal fluorescence imaging of Rab5 and pEGFR at the apical plasma membrane in cells treated with EGF-Flat. Scale bars: 5 μm.
